# Multifunctional Chemical Sensing Platform Based on Dual‐Resonant Infrared Plasmonic Perfect Absorber for On‐Chip Detection of Poly(ethyl cyanoacrylate)

**DOI:** 10.1002/advs.202101879

**Published:** 2021-08-22

**Authors:** Dongxiao Li, Hong Zhou, Xindan Hui, Xianming He, He Huang, Jiajia Zhang, Xiaojing Mu, Chengkuo Lee, Ya Yang

**Affiliations:** ^1^ Key Laboratory of Optoelectronic Technology & Systems of Ministry of Education International R & D center of Micro‐nano Systems and New Materials Technology Chongqing University Chongqing 400044 P. R. China; ^2^ CAS Center for Excellence in Nanoscience Beijing Key Laboratory of Micro‐nano Energy and Sensor Beijing Institute of Nanoenergy and Nanosystems Chinese Academy of Sciences Beijing 100083 P. R. China; ^3^ Department of Electrical and Computer Engineering Center for Intelligent Sensors and MEMS (CISM) and NUS Graduate School for Integrative Sciences and Engineering National University of Singapore Singapore 117576 Singapore; ^4^ School of Nanoscience and Technology University of Chinese Academy of Sciences Beijing 100049 P. R. China; ^5^ Center on Nanoenergy Research School of Physical Science and Technology Guangxi University Nanning 530004 P. R. China

**Keywords:** infrared spectroscopy, metamaterial absorbers, multifunctional sensors, poly(ethyl cyanoacrylate) sensing, surface‐enhanced infrared absorption

## Abstract

Multifunctional chemical sensing is highly desirable in industry, agriculture, and environmental sciences, but remains challenging due to the diversity of chemical substances and reactions. Surface‐enhanced infrared absorption (SEIRA) spectroscopy can potentially address the above problems by ultra‐sensitive detection of molecular fingerprint vibrations. Here, a multifunctional chemical sensing platform based on dual‐resonant SEIRA device for sensitive and multifunctional on‐chip detection of poly(ethyl cyanoacrylate) (PECA) is reported. It is experimentally demonstrated that the SEIRA sensing platform achieves multiple functions required by the PECA glue industry, including vibrational detection, thickness measurement, and in situ observation of polymerization and curing, which are usually realized by separately using a spectrometer, a viscometer, and an ellipsometer in the past. Specifically, the all‐in‐one sensor offers a dual‐band fingerprint vibration identification, sub‐nm level detection limit, and ultrahigh sensitivity of 0.76%/nm in thickness measurement, and second‐level resolution in real‐time observation of polymerization and curing. This work not only provides a valuable toolkit for ultra‐sensitive and multifunctional on‐chip detection of PECA, but also gives new insights into the SEIRA technology for multi‐band, multi‐functional, and on‐chip chemical sensing.

## Graphical abstract

A chemical sensing platform based on dual‐resonant surface‐enhanced infrared absorption (SEIRA) effect is developed for sensitive and multifunctional on‐chip detection of poly(ethyl cyanoacrylate), including vibrational detection, thickness measurement, and in situ observation of polymerization and curing. This work provides a valuable toolkit for glue industry, as well as, pushing forward the frontier of SEIRA technology to multifunctional chemical sensing.

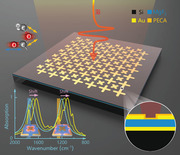

## 1 Introduction

Detection of chemicals plays a fundamental role in industry, agriculture, and environmental sciences. Sensing methods with the characteristics of multi‐function, high sensitivity, and low cost are ideal, and their development requires advanced technology coupled with basic knowledge in chemistry and material sciences.^[^ ^1^ ^]^ In recent years, surface‐enhanced vibrational spectroscopy has been widely used in the detection of chemicals and has the potential to meet these requirements.^[^ ^2^, ^3^, ^4^, ^5^ ^]^ As an indispensable branch, surface‐enhanced infrared absorption (SEIRA) has made significant progress in both theory and experiment, and has given rise to a huge number of applications, such as, imaging of hyperspectral infrared chemistry,^[^ ^6^, ^7^ ^]^ probing of plasmonic near‐field,^[^ ^8^, ^9^ ^]^ ultra‐sensitive detection of chemicals and molecular substances,^[^ ^10^, ^11^, ^12^, ^13^ ^]^ and monitoring of dynamic processes.^[^ ^14^, ^15^ ^]^ Among them, the implementation of chemical sensing applications is based on the strategy of placing the target molecular in the near‐field (hotspots) of nanostructures, where the near‐field intensity is strengthened by several orders of magnitude due to the surface plasmonic effect of nanostructures.^[^ ^16^ ^]^ Metamaterial perfect absorbers (MPAs) are an excellent candidate for SEIRA spectroscopy applications due to its ability to create stronger near‐field enhancements than metasurfaces.^[^ ^17^ ^]^ A well‐designed MPA can generate such intense highly confined hotspots in the mid‐infrared range, which is the fingerprint region of many chemical molecules.^[^ ^18^ ^]^ Strong interaction between hotspots and chemical molecules greatly enhances the fingerprint vibrations of chemicals, thereby realizing highly sensitive detection of trace amounts of chemical molecules and real‐time monitoring of dynamic processes.

However, molecular fingerprints are often composed of multiple characteristic infrared (IR) absorbance peaks, and single‐band MPAs are limited by the number of bands of hotspots and cannot adequately detect molecular fingerprints.^[^ ^19^ ^]^ Therefore, the development of MPAs with dual‐band or even multi‐band have become a hot spot in spectroscopy research, and many efforts have been invested for it, such as, asymmetric cross‐shape arrays,^[^ ^20^, ^21^ ^]^ single‐sized nanodisk arrays,^[^ ^22^, ^23^ ^]^ and uniform arrays of multi‐resonant elements.^[^ ^24^, ^25^ ^]^ These methods of using the second or third order of the basic magnetic mode to achieve multi‐band MPAs reduce efficiency levels of higher‐order modes, resulting in the degradation of SEIRA performance.^[^ ^26^, ^27^ ^]^ Moreover, the electromagnetic coupling between different resonance modes hinders the direct spectral tuning of each resonance.^[^ ^28^ ^]^ Therefore, high‐efficiency, independently adjustable, polarization‐insensitive methods for multi‐band MPAs is still of great significance to SEIRA spectroscopy. Beyond that, the coupling between molecules and plasmonic hotspots is critical to molecular detection. The main factors affecting coupling efficiency include i) the coupling strength,^[^ ^29^ ^]^ ii) the spatial position of the molecule in the plasmonic near‐fields,^[^ ^30^ ^]^ and iii) the spectral detuning between the molecular vibration and the plasmonic resonance.^[^ ^31^ ^]^ These scientific issues are very significant in single‐band MPAs,^[^ ^32^ ^]^ which are more complicated in dual‐band or multi‐band MPAs due to the existence of interference between different plasmonic resonances. Therefore, the use of multi‐band MPAs for highly sensitive and efficient sensing applications still presents a challenge.

From the perspective of chemical sensing, the diversity of chemicals and chemical reactions determines that the methods and instruments for the measurement of multiple chemical parameters and reactions is diverse and complex. For instance, the identification of chemical substances is generally realized by detecting the fingerprint of chemicals using an infrared spectrometer.^[^ ^33^ ^]^ The observation of the chemical polymerization reaction is usually achieved by determining the viscosity of chemicals with a viscometer.^[^ ^34^ ^]^ The film thickness of the chemical is often obtained by measuring the amplitude and phase of light in chemicals using an ellipsometer.^[^ ^35^ ^]^ Although these methods achieve the measurement of various parameters of chemicals, the disadvantages of low efficiency, high cost, and complicated operation are not negligible, especially when multiple chemical parameters are measured simultaneously. Therefore, multifunctional, simple, and low‐cost methods for the sensing of multiple parameters and reactions of chemicals are highly desired.

**Figure 1:**
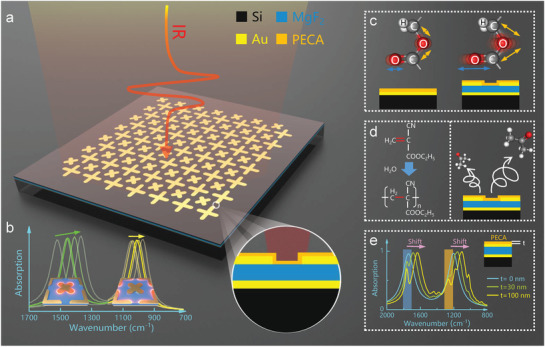
Illustration of multifunctional chemical sensing platform for on‐chip detection of poly(ethyl cyanoacrylate). a) 3D schematic view of the dual‐resonant MPAs sensing platform. b) Individual resonance tuning in a dual‐resonant array by adjusting the length of the corresponding antenna. c) Vibrational sensing of the PECA, d) in situ observation of ECA polymerization and precipitation, and e) thickness measurement of PECA by using multifunctional MPA‐based sensing platform.

In this work, we present a multifunctional chemical sensing platform based on dual‐resonant infrared plasmonic MPA for sensitive and multifunctional on‐chip detection of poly(ethyl cyanoacrylate) (PECA) (**Figure** **1**), which are critical in research and development of the glue industry. The dual‐resonant plasmonic MPA is implemented by the strategy of allocating different resonances on multiple overlapping arrays with the same period to overcome the two major obstacles mentioned above: i) SEIRA performance degradation caused by multiple resonances and ii) electromagnetic coupling between different resonance modes. Investigations in the later section reveal that the dual‐resonant MPA is highly efficient, resonance adjustable, and polarization‐insensitive. Then, the multifunctional MPA‐based platform is exploited to comprehensively sense PECA, including vibrational detection of PECA, in situ observation of the PECA polymerization and curing, and thickness measurement of PECA. The vibrational detection of PECA molecules is achieved by matching its resonances with the two vibrational bands of PECA (stretching absorption C = O at 1747.5 cm^–1^ and C—O—C at 1252.8 cm^−1^) (Figure 1c).^[^ ^36^ ^]^ In situ observation of the PECA polymerization and curing is realized by using the MPA to monitor the change of the bond accompanying the reaction (Figure 1d). Thickness measurement is based on the redshift and enhanced absorption of MPA caused by thickness changes (Figure 1e). With the advantage of multifunction, MPA‐based chemical platform allows multiple parameters and reactions of PECA to be measured on one chip instead of separately using traditional spectroscopy, viscometer, and ellipsometer, respectively, which makes chemical detection simple, low‐cost, and efficient. Finally, we revised general equations of the coupled‐mode theory modeling the plasmonic sensing system to improve the model accuracy, and further comprehensively analyzed the significant parameters affecting coupling through experiments. In short, this work will mainly focus on the detailed study of dual‐resonant infrared plasmonic perfect absorber, its multifunctional application in vibrational detection, in situ observation of reactions, and thickness measurement. We believe the findings will gain new insights into multifunctional on‐chip chemical detection based on SEIRA technology and promote the development of chemical discipline in terms of efficiency, speed and multifunction.

## 2 Results and Discussions

### 2.1 Design and Optical Properties of Dual‐Resonant Metamaterial Perfect Absorbers

**Figure 2:**
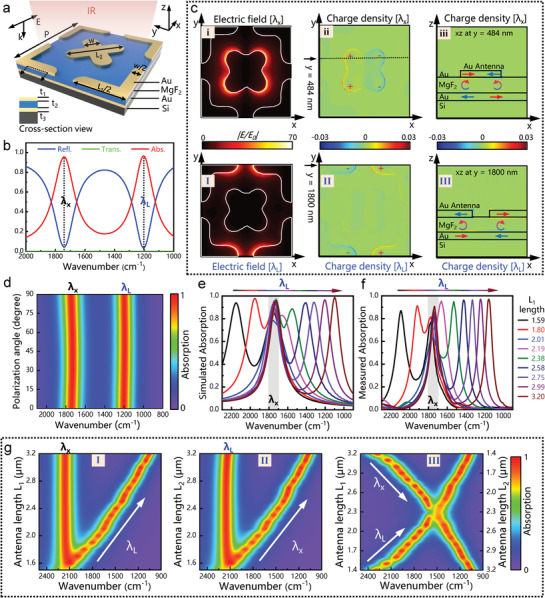
Design and optical properties of dual‐resonant MPAs. a) Schematic illustration of the dual‐resonant metamaterial perfect absorber (MPA). Dimensions: *L*_1_ = 2.99 µm, *L*_2_ = 2.05 µm, *W* = 750 nm, *t*_1_ = 100 nm, *t*_2_ = 200 nm, *t*_3_ = 100 nm, *P* = 3.6 µm. b) The simulated spectra of the dual‐resonant MPA with two perfect absorption peaks at 1740 cm^–1^ (*λ*_X_) and 1200 cm^–1^ (*λ*_L_). c) The electric field and charge distribution of the dual‐resonant MPA at *λ*_X_ and *λ*_L_ resonances shown in (b). I, II, i, ii: Field distribution calculated at the top surface of the nanostructure. III, iii: Cross‐sectional view of the charge distribution. Arrows indicate the direction of the current flow. d) The simulated spectra of the MPA with different polarizations, investigating its polarization‐insensitive characteristics. e) Simulated and f) measured spectra of dual‐resonant MPA with different *L*_1_ and the same period (3.6 µm). g) Contour mapping of simulated absorption spectra of dual‐resonant MPA when changing I) *L*_1_ only, II) *L*_2_ only, III) *L*_1_ and *L*_2_ simultaneously.

The proposed dual‐resonant MPA, as illustrated in **Figure** **2**a, is composed of a gold ground layer, a magnesium fluoride (MgF_2_) dielectric spacer, a gold nanofabricated metamaterial structure, and a silicon substrate. In this tri‐layer sandwich‐like configuration, the incident IR light is trapped and absorbed due to the interference between the multiple reflections in the cavity. By reasonably optimizing the layer thickness and the top cross‐shaped metamaterial pattern, perfect absorption can be achieved at resonance (Figure 2b). The top metamaterial pattern is the key to the MPA to obtain multiple resonances. The strategy we propose here to achieve dual resonance is to rotate two arrays with the same period by a certain angle (45° in this work) and then combine them (Figure S1, Supporting Information). Figure 2a displays the unit cell of the dual‐resonant MPA, its resonances can be individually adjusted to suit different applications by changing the length of the pattern, which will be further analyzed later. Our pattern rotation strategy exhibits many advantages: i) More space and freedom for the pattern to adjust the length, due to the staggered layout created by rotation (Figure S1, Supporting Information); ii) easier to extend to multi‐resonant MPA because of the space advantage. The method is scalable to achieve multi‐resonant MPA by integrating new elements into the available space when required by applications (Figure S2, Supporting Information); iii) achieving near‐unit absorption at all resonances, which is due to that all cross‐shaped antenna work in the first‐order resonance mode.

In order to investigate the mechanism of dual resonance, the optical response of dual‐resonant MPA was simulated and analyzed using finite‐difference time‐domain (FDTD) software. Figure 2c shows the distribution of the local enhanced electric fields (*E*/*E*_0_) and charge density of dual‐resonant MPA at *λ*_X_ and *λ*_L_. It can be seen from the local enhanced electric fields distribution (Figure 2c‐i,c‐I): i) The local electric field is mainly concentrated on the tip ends of the pattern along the polarization direction and creates an electric dipole, which is consistent with the tip effect reported previously; ii) The local enhanced electric fields corresponding to *λ*_X_ and *λ*_L_ are located on the X‐shaped and L‐shaped patterns respectively, which reveals that the two resonances are independent in space and have little interference with each other. Similar to the distribution of the electric field, the charge on the surface of the pattern is also concentrated on the tip ends of the pattern (Figure 2c ‐ii,c‐II). Besides, the induced charges between the Au antenna and the Au ground form an antiparallel current, thereby creating a magnetic dipole, as shown in Figure 2c ‐iii,c‐III. The effective permittivity and permeability of the MPA can be adjusted by electric and magnetic dipoles created by polarization and current loops. When the impedance ($Z=\sqrt{\mu /\varepsilon }$) of the MPA matches the free space, reflection is minimized and the transmittance is nearly zero due to the presence of the Au ground plane. Finally, near‐unity absorption is realized at both *λ*_X_ and *λ*_L_ (Figure 2b). Additionally, the well‐designed MPA shows polarization‐insensitive characteristics because of the structural symmetry (Figure 2d).

Importantly, the MPAs exhibit geometry‐dependent characteristics, that is, its resonance can be adjusted by designing its geometric dimensions. For demonstration purposes, we fabricated MPA arrays with different length *L*_1_ (Figure S3, Supporting Information), and their simulated and measured spectra are shown in Figure 2e,f. Here, the dimension parameter of the antenna used in the simulation is 1:1 extracted from the SEM antenna topography (Figure S4, Supporting Information). The following essential findings were obtained by comparing these results: i) The measured spectra were consistent with the simulated spectra, and the absorption intensity of MPA at resonance was within 0.9–0.98; ii) the resonance *λ*_L_ red‐shifted as *L*_1_ increases, while the resonance *λ*_X_ remained unchanged due to the constant dimension of *L*_2_. It confirms the conclusion from the previous field simulation that the two resonances are weakly coupled and their electromagnetic responses are independent of each other. Furthermore, we investigated the spectral response of dual‐resonant MPAs when changing *L*_1_ only (Figure 2g‐I), ii) changing *L*_2_ only (Figure 2g ‐II), and changing *L*_1_ and *L*_2_ simultaneously (Figure 2g ‐III) through simulations. The results revealed that the two resonances were adjustable and determined by the corresponding pattern dimension. This independence is a critical characteristic, which allows the resonance of the MPA to be adjusted in a simple manner, thereby enhancing the vibration of the analyte of interest.

### 2.2 Material Characterization of Multifunctional Metamaterial Perfect Absorber‐Based Sensing Platform

**Figure 3:**
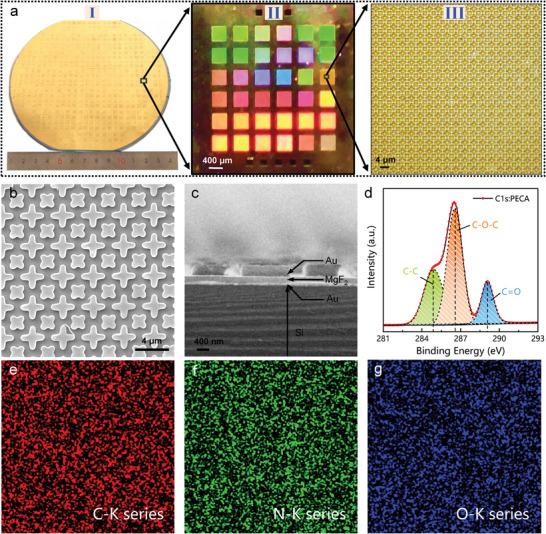
Characterization of multifunctional MPA‐based sensing platform. a) Optical photograph of the MPA‐based sensing platform. b) SEM micrograph showing top and (c) cross‐sectional views of device. d) High‐energy‐resolution XPS spectrum showing the main chemical bonds of PECA. e–g) EDS mappings of C, N, and O elements in PECA showing the details of coating distribution on sensing platform.

The dual‐resonant MPA was fabricated by stepping UV lithography, electron beam evaporation and etching (see Section 4 for details). The optical photograph of the fabricated MPA are shown in **Figure** **3**a. Clearly, 36 MPA arrays were integrated on a 5 mm^2^ × 5 mm^2^ chip (Figure 3a ‐II), and nearly 500 such chips were fabricated on a 6‐inch silicon wafer, which means that nearly 18 000 dual‐resonant MPAs could be obtained in one fabrication. The large number greatly reduced the cost of the device, making the technology more competitive in sensing applications. In addition, the top view of scanning electron microscope (SEM) image reveals that the fabricated MPA had well‐defined periodic structures (Figure 3b), and the cross‐sectional view indicates that Au/MgF_2_/Au layer adheres tightly to the Si layer (Figure 3c), which was the basis for the agreement between the experimental results and the simulation results. Figure 3d shows the high‐energy‐resolution X‐ray photoelectron spectroscope (XPS) spectrum of PECA. As observed, the relative content of C—O—C bonds is higher than that of C—C and C = O, which is in agreement with the data in the previously reported literature.^[^ ^37^ ^]^ The energy dispersive X‐ray spectroscopy (EDS) result in Figure 3e,f and Figure S5, Supporting Information, verified the presence of C, N, and O in PECA, and the distribution of each element was relatively uniform in the device surface. Collectively, all of the above morphological and compositional analyses demonstrated the successful fabrication MPA devices and the uniform growth of PECA films.

### 2.3 Vibrational Sensing of the Poly(ethyl cyanoacrylate) Using Multifunctional Metamaterial Perfect Absorber‐Based Sensing Platform

**Figure 4:**
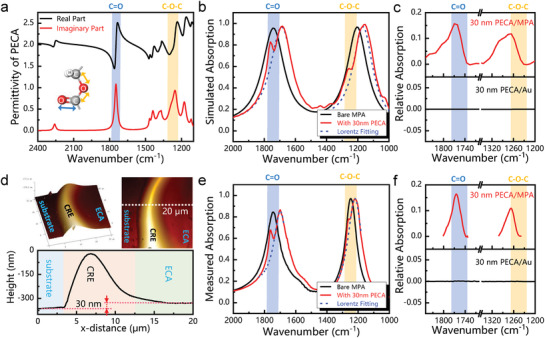
Vibrational sensing of the PECA using multifunctional MPA‐based sensing platform. a) The real and imaginary parts of the composite permittivity of PECA in the mid‐infrared region. The two vibrations of PECA are located at 1747.5 cm^–1^ (C = O) and 1252.8 cm^–1^ (C—O—C). b) The simulated spectra of the dual‐resonant MPA before and after covered with 30 nm PECA film, showing the details of detecting the two vibrations of PECA simultaneously. Black curve: bare MPA, Red solid line: MPA with PECA, Blue dashed line: baseline. c) Relative absorption spectra of PECA extracted from (b) reflecting the intensities of the two vibrations of PECA measured on Au and MPA. d) Thickness measurement of the PECA by using AFM method. e,f) Measured results corresponding to the simulated results in (b) and (c).

Ethyl cyanoacrylate (ECA) is an ethyl ester of 2‐cyano‐2‐propenoic acid, and widely used as an adhesive in the industry due to its rapid polymerization reaction to form PECA. According to its infrared spectrum (Figure S6, Table S1, Supporting Information), there are two strong vibrations in the mid‐infrared range, namely C = O at 1747.5 cm^−1^ and C—O—C at 1252.8 cm^−1^. Therefore, it is very suitable as an analyte to investigate the SEIRA sensing capability of the dual‐resonant MPA. **Figure** **4**a shows the complex permittivity of PECA in the mid‐infrared region obtained by using the Lorentz resonance model (Figure S7, Table S2, Supporting Information). By importing it into the FDTD software, the simulated spectrum of the MPA covered with a 30 nm thick PECA film were calculated, as shown in Figure 4b. Clearly, the presence of PECA caused a redshift of the resonance of the MPA, which was attributed to the dielectric interference of PECA on the plasmonic resonance.^[^ ^38^ ^]^ Furthermore, the vibrations at 1747.5 cm^−1^ (C = O) and 1252.8 cm^−1^ (C—O—C) of PECA caused obvious changes in the lineshape of the spectrum and were well matched with *λ*_X_ and *λ*_L_ resonant mode, respectively. To quantitatively analyze the change of the lineshape, the Lorentz model was applied to calculate the baseline of the spectrum, which was then used as a reference for transformation to extract the enhanced spectra of PECA vibrations. The relative absorption spectrum was calculated as △*A* = log_10_ (*A*_PECA_/*A*_fit_), as shown in Figure 4c, and for comparison, the spectra of Au mirrors covered with 30 nm thick PECA were also measured by Fourier transform infrared (FTIR) spectrometer with the same setting. No obvious signal were observed in the relative absorption spectrum of the 30 nm PECA/Au configuration (black line). However, the vibration signals of C = O and C—O—C were very distinct in the extracted spectrum at 30 nm PECA/MPA configuration (red line), which is attributed to the enhancement effect of SEIRA.

Further, we demonstrate the simulated results through experiments (Figure 4d–f). Prior to the SEIRA measurement, an atomic force microscope (AFM) measurement was carried out to determine the thickness of the PECA film. The sample was pre‐treated with acetone before measurement: the PECA covering the surface of the device was partially dissolved and washed away by acetone, while the unwashed PECA remained on the surface, which would form a height difference on the substrate. The height difference was then detected by AFM. Figure 4d shows the measured results of PECA film formed at a rotation speed of 6000 rpm and a mass ratio of 1:6 (ECA: acetone). Clearly, the height difference between the upper surface of the PECA and the substrate is 30 nm, which is considered to be the thickness of the PECA on the device. Notably, there is an area caused by coffee‐ring effect (CRE) in the transition area between PECA and the bare substrate. The height of CRE exceeds 300 nm, which is caused by the expansion and agglomeration of PECA in the transition area when acetone dissolves PECA. Figure 4e,f describe the experimental results of using the dual‐resonant MPA to detect PECA film, which are consistent with the simulation results, verifying the correctness of the previous discussion. To quantitatively evaluate the enhancement effect, the enhancement factor (EF) of the plasmonic MPA is defined as:

(1) $$\mathrm{EF}=\frac{I_{\mathrm{SEIRA}}}{I_{\mathrm{ref}}}\frac{N_{\mathrm{ref}}}{N_{\mathrm{SEIRA}}}$$

where *I*_SEIRA_ and *I*_ref_ are intensities of the molecular vibration of PECA. *N*_ref_ and *N*_SEIRA_ are the number of PECA molecules contributed to vibrational signals (Figure S8, Supporting Information). It can be obtained by calculation that the EF of the two vibrations of 30 nm thick PECA at *λ*_X_ and *λ*_L_ are 449 and 334, respectively. In addition, the proposed MPA has potential for chemical identification of trace amounts of molecules (Figure S9, Supporting Information).

### 2.4 In Situ Observation of Ethyl Cyanoacrylate Polymerization and Precipitation Using Multifunctional Metamaterial Perfect Absorber‐Based Sensing Platform

**Figure 5:**
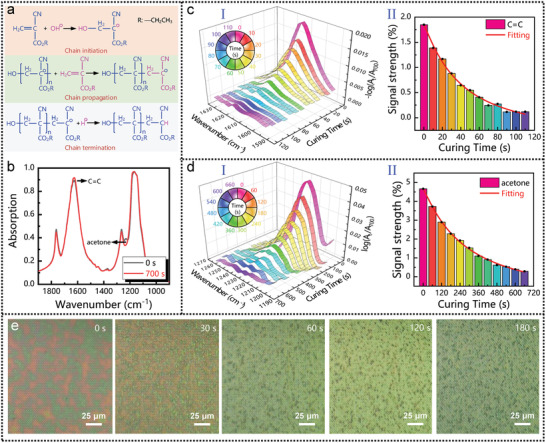
In situ observation of ECA polymerization and precipitation using multifunctional MPA‐based sensing platform. a) Schematic illustration of the ECA polymerization reaction. b) Spectral changes caused by ECA polymerization and PECA curing. The vibration intensity of the C = C bond is used as the indicator of the progress of the polymerization reaction, and the absorption at 1221 cm^–1^ represents the progress of the PECA curing reaction. The evolution of spectra as a function of the c) polymerization time and d) curing time. I: relative absorption spectra; II: the maximum value of the relative absorption. e) Optical photograph showing the process of polymerization and curing reactions.

In situ observation of dynamic chemical reactions is another function of our multifunctional MPA‐based sensing platform, which is an important means for investigating the characteristics of chemical reactions in chemical synthesis and molecular dynamics.^[^ ^39^ ^]^ As a proof of concept, the device was used to monitor the polymerization reaction of 100 nm thick ECA, as well as its curing from the organic solvent (acetone) after polymerization. **Figure** **5**a shows the ECA polymerization process. In the presence of trace water molecules, the hydroxide ions (OH—) initiate the polymerization by breaking the C = C bond and forming anions, which are stabilized by electron‐withdrawing —COOC_2_H_5_ and —CN groups.^[^ ^40^ ^]^ The polymerization of ECA continues through the propagation reaction until it is completely formed into PECA. The whole process is accompanied by the breaking of the C = C bond, so we leverage our dual‐resonant MPA to detect the change in C = C vibration to achieve real‐time monitoring of the process. *λ*_X_ resonance is chosen as the working mode for real‐time monitoring due to its close proximity to the vibration of the C = C bond (1614 cm^−1^), as shown in Figure 5b. Notably, a decrease in the spectral intensity at 1747 cm^−1^ corresponding to C = O in PECA was observed. It is due to that, the vibration of C = O bonds is effected by the structural changes of ECA molecules during the polymerization reaction. The experiment started with the loading of the ECA onto the MPA. Then, the spectrum of the MPA was continuously measured and recorded while the polymerization reaction occurred. Figure 5c‐I depicts the relative absorption of C = C bond as a function of polymerization time, and the maximum value of the relative absorption is used as the signal intensity representing the bond change, as shown in Figure 5c ‐II. Clearly, the signal intensity of C = C bond decreased significantly with the increase of polymerization time, and then disappeared completely after about 120 s, indicating that the polymerization reaction was complete. The signal intensity reduced sharply in the first 20 s, which was due to the rapid polymerization of ECA on the air‐contacting surface. The subsequent stage of gradual weakening was attributed to the fact that the small amount of internal ECA close to the device polymerized slowly in the absence of hydroxide ions.

Apart from the monitoring of chemical reactions, the curing of the PECA from acetone is also investigated. Generally, when added to a solvent, PECA sequentially undergoes a swelling stage in which solvent molecules diffuse into polymer molecules, and a dissolution stage in which polymer molecules diffuse into the solvent.^[^ ^41^, ^42^ ^]^ The curing of PECA we investigated here is the opposite process, in which solvent molecules escape from PECA molecules. This process is spectrally manifested as a decrease in absorption near 1221 cm^−1^ (asymmetric stretching vibration of the acetone molecule), as shown in Figure 5b. *λ*_L_ resonance is chosen as the working mode for real‐time monitoring of PECA curing due to its close proximity to asymmetric stretching vibration of the acetone at 1221 cm^−1^. The relative absorption and the corresponding signal strength at 1221 cm^−1^ as a function of curing time are plotted in Figure 5d ‐I,d‐II. Clearly, the signal intensity of vibration of the acetone molecules at 1221 cm^−1^ decreased significantly with the increase of curing time, and then disappeared completely after about 10 min, indicating that the curing was completed (Figure 5d ‐II). Compared with the polymerization of PECA, the curing signal intensity changed more slowly, indicating that the escape speed of acetone molecules from PECA is slower than the PECA polymerization speed. Notably, in the absence of our MPA, only when the thickness is equal to or greater than 30 nm, the vibration signal is strong enough to monitor polymerization and curing. It reveals the role of MPA in sensing: i) The near field of MPA effectively enhances the molecular vibration intensity of ultra‐thin analytes; ii) MPA has the ability to selectively enhance the target vibration, thereby reducing the interference of the background scanning and environment on the absorption spectrum of PECA, that is, noise signals outside the target vibration will not be amplified. Figure 5e shows the optical photos of PECA on the device at different times. As observed, part of the ECA has already polymerized at the beginning of the measurement, which is caused by the delay between the spin‐coating and the beginning of FTIR measurement. The delay time is about 5 s, and the beginning of FTIR measurement is defined as 0 s. Then, the morphology of the analyte on the surface of the device changed significantly in the first 30 s due to the violent polymerization reaction of ECA. After 60 s, no obvious changes were observed in the optical photos, indicating that this method cannot detect the subtle change phase of the chemical reaction. Fortunately, SEIRA provides an effective method for monitoring the chemical reaction process with higher detection level. Collectively, our proposed MPA realizes the process monitoring of chemical polymerization reaction and physical curing, which is of great significance in the field of analytical chemistry and physical reaction monitoring.

### 2.5 Thickness Measurement of Poly(ethyl cyanoacrylate) Using Multifunctional Metamaterial Perfect Absorber‐Based Sensing Platform

**Figure 6:**
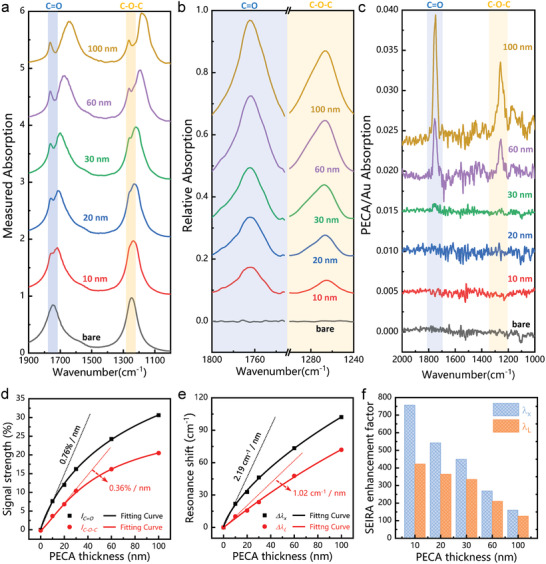
Thickness measurement of PECA using multifunctional MPA‐based sensing platform. a) The absorption spectra of MPA covered with different thicknesses of PECA. The thickness varies from 10 to 100 nm. b) The corresponding relative absorption spectrum. c) Absorption spectra of PECA covered on a gold mirror measured by normal incidence. d) The evolution of signal strength as a function of PECA film thickness. e) The evolution of resonance shift as a function of PECA film thickness. f) SEIRA enhancement factor as a function of PECA film thickness obtained from measurements.

In this section, we experimentally study thickness measurement of PECA using multifunctional MPA‐based sensing platform. The thickness of PECA is controlled by the concentration of ECA and the spin‐coating speed (see method for details). **Figure** **6**a describes the measured absorption spectra of the MPA when the thickness of PECA increases from 0 to 100 nm. With the increase of the PECA thickness, a distinct resonance wavelength redshift is observed on the spectra of the MPA, while both vibrations of the PECA molecule are fixed. The mismatch between the redshift of resonance wavelength and the fixation of molecule vibration gives rise to the concept of detuning. The detuning is defined as the wavelength difference between vibration and resonance (*λ*_X,L_ – *λ*_C = O,C—O—C_), where *λ*_X,L_ and *λ*_C = O,C—O—C_ are the wavelengths of resonance and vibration, respectively. As observed, the detuning becomes larger as the thickness increases, and the detuning is nearly zero when the thickness of PECA is small. After extracting the relative absorption spectrum from the spectrum (see Section 4 for details), it can be seen that the vibration signal intensity of C = O and C—O—C becomes stronger with the increases of PECA thickness (Figure 6b). For comparison, the absorption spectrum of PECA on a gold mirror was measured at normal incidence (Figure 6c). Clearly, in the PECA/Au configuration, weak vibration is observed only when the thickness of PECA is equal to or greater than 30 nm. However, by using SEIRA device, the vibration of 10 nm thick PECA can also be observed, which benefits from the SEIRA enhancement of the device. In addition, we estimate that the detection limit of the device is at the sub‐nm level due to the 4 cm^−1^ resolution of the experimental platform.

Furthermore, the evolution of signal strength, resonance shift and SEIRA *EF* as a function of PECA film thickness is obtained, as shown in Figure 6d,e. The signal strength rises sharply with the increase of PECA thickness, and then saturates in the thicker layer (Figure 6d), which is determined by the near‐field distribution of the plasmonic SEIRA device. To prove this hypothesis, the spatial profile of the near‐field electric field intensity along the direction perpendicular to the Au structure surface was calculated by using FDTD simulations (Figure S10, Supporting Information). The results reveal that the near‐field intensity attenuates sharply as it moves away from the gold surface, and decays to 10% around 220 nm, which is defined as the penetration depth. Moreover, the detection sensitivity of C = O (0.76%/nm, 2.19 cm^−1^/nm) is higher than that of C—O—C (0.36%/nm, 1.02 cm^−1^/nm), which is related to their own vibration intensity and the coupling with near field. In order to better understand the influence of the spatial distribution of the near‐field intensity on the signals strength of the analyte, we calculated and obtained the relationship between the EF and the thickness of the PECA film (Figure 6f). The result shows that the molecular vibrations located near the surface of the nanostructure are significantly enhanced, while the enhancement of molecular vibrations farther from the surface are weaker. In addition, we have repeatedly recorded the background noise of the device (less than ±0.25%), and the intensity of the background noise is less than 0.76%/nm (C = O) and 0.36%/nm (C—O—C) (Figure S11, Supporting Information). Therefore, we evaluate that the MPA sensing platform has sub‐nanometer detection sensitivity. The evolution of resonance shift (Figure 6e) in SEIRA device is similar to the enhancement behavior of molecular vibration, and it also undergoes a process of a sharp increase and then a gradual saturation, which indicates that the shift of the resonance is also related to the position of the molecule in the near field. In addition, the simulation results are in good agreement with the experimental data (Figure S12, Supporting Information), and the slight discrepancy may be caused by fabrication errors and simulation modeling. The above investigation shows that the proposed MPA can be used as a powerful tool for thickness sensing of ultra‐thin films.

To understand the mechanism of the above behavior, we use the temporal coupled mode theory (TCMT) for theoretical analysis. The detailed coupled modal derivation equation is provided in Note S13, Supporting Information. The modified absorption of the PECA‐coated MPA platform can be described by the following equation:^[^ ^43^ ^]^

(2) $$\begin{array}{c} A_{3}=1-\left|1-\frac{2\gamma_{1e}}{j\left(\omega -\omega_{1}+\Delta \omega_{1}\right)+\gamma_{1o}+\gamma_{1e}+\Gamma_{b}}\right.\\ {\left.-\frac{2\gamma_{2e}}{j\left(\omega -\omega_{2}+\Delta \omega_{2}\right)+\gamma_{2o}+\gamma_{2e}+\Gamma_{c}}\right|}^{2} \end{array}$$

where *γ*_1o,2o_ and *γ*_1e,2e_ are the internal absorption loss rate and the external radiation loss rate of the harmonic oscillator, respectively, *ω*_1_ and *ω*_2_ are the center frequencies of the metamaterial resonator, and Δ*ω*_i_ is a correction term, which describes the redshift caused by the analyte.

(3) $$\Gamma_{b}=\mu_{1b}^{2}/\left[j\left(\omega -\omega_{b}\right)+\gamma_{b}\right]$$

and

(4) $$\Gamma_{c}=\mu_{2c}^{2}/\left[j\left(\omega -\omega_{c}\right)+\gamma_{c}\right]$$

are the vibration modes of C = O bond and C—O—C bond in PECA molecule, respectively. The parameters *ω*_b_(*ω*_c_), *γ*_b_(*γ*_c_) are the center frequency and absorption damping of C = O (C—O—C) respectively, *μ*_1b_ (*μ*_2c_) is the coupling rate between C = O (C—O—C) vibration and metamaterial resonator. Figure S14c, Supporting Information, describes the fitting result of the measured spectral data calculated by using modified model, and an excellent fit was observed, which proves the validity of the modified model. Then, the modified model is used to further analyze the coupling between PECA and the MPA. The measured spectra of the MPA covered with different thicknesses of PECA are fitted through the modified model, and the corresponding coupling parameters, including coupling coefficient, damping, radiation damping and loss damping, are extracted (Note S13, Figure S14, Table S3, Supporting Information). In particular, both the coupling coefficient *µ*_1b,2c_ and damping *γ*_b,c_ of PECA rise with the increase of the PECA thickness. Since thicker PECA means more molecules in the near field of the MPA and the PECA molecules are purely dissipative modes in the coupling system, the increase in thickness results in greater damping and stronger coupling. By contrast, non‐radiative (*γ*_1o,2o_) and radiative damping rates (*γ*_1e,2e_) are almost constant with the increase of analyte thickness. This is because after the device is fabricated, *γ*_1o,2o_ and *γ*_1e,2e_ of the device are determined accordingly, and are not affected by the thickness of the analyte. In addition, the redshift of the resonant frequency is positively correlated with the PECA thickness (Figure S14f, Supporting Information), which is consistent with the result in Figure 6e. This correlation indicates that the shift of the resonance frequency can be used as an indicator for the quantitative detection of trace amount of analytes. It should be emphasized that the consideration of redshift in the TCMT model has never been done before, and it is very important. Its importance includes two aspects: 1) Considering that the redshift of TCMT can distinguish the initial state of the device and the state after adding the sample, this modification makes the TCMT model more rigorous. 2) Considering the red shift can add a new physical quantity (Δ*ω*) to the TCMT model. In fact, this physical quantity exists objectively, and it is related to the real part of the complex dielectric function of the sample to be analyzed. The consideration of the amount of redshift will bring new perspectives and vitality to the TCMT model, which is expected to further guide the design of the device theoretically.

## 3 Conclusion

In summary, we have demonstrated a multifunctional chemical sensing platform based on dual‐resonant infrared plasmonic MPA for sensitive on‐chip detection of multiple parameters and reactions of PECA, including vibrational detection, in situ observation of polymerization and curing, and thickness measurement. The fabricated dual‐resonant MPA achieves an absorption higher than 90% at both resonances, and the two resonances can simultaneously detect the vibration of the C = C and C—O—C bonds in PECA, thereby realizing the in situ observation of the ECA polymerization and the PECA curing. Due to the near‐field intensity enhancement of MPA, it breaks through the detection limit of the conventional FTIR methods and achieve sub‐nm level detection limit. In theory, the TCMT‐based model is modified to improve accuracy, and then the modified model is utilized to comprehensively analyze the important parameters affecting the coupling system. This work not only provides a powerful tool for on‐chip detection of multiple parameters and reactions of PECA, but also gains new insights into multifunctional chemical detection using dual‐resonant infrared plasmonic MPA.

## 4 Experimental Section

### Numerical Simulations

Numerical calculations were performed using commercial software packages (FDTD Solutions ver. 8.19, Lumerical). The coordinate parameters of the antenna are derived from the morphology of the antenna observed by the SEM, and the 1:1 antenna modeling has been completed in the FDTD simulation software. To improve simulation efficiency, periodic boundary conditions were adopted along the x and y directions, and perfectly matched layer boundary conditions were used along the *z* direction. The refractive index of MgF_2_ is set to 1.38, and the complex refractive index of gold was taken from palik et al. The dielectric function of PECA was obtained based on the infrared absorption spectrum and the Lorentz resonance model. A 10 nm thick Ti adhesion layer was omitted in the simulation.

### Device Fabrication

Complementary metal‐oxide‐semiconductor compatible processes were used to fabricate the dual‐resonant MPA. The detailed fabrication process is as follows: i) Fabrication began with the cleaning and drying of a high‐resistivity 6" silicon wafer; ii) 10 nm thick Ti and 100 nm thick Au films were sequentially deposited on the Si wafer by magnetron sputtering, where Ti acted as an adhesion layer to enhance the adhesion between the gold film and the Si substrate; iii) a MgF_2_ layer with a thickness of 200 nm was deposited on the gold film by electron beam evaporation; iv) 10 nm Ti and 100 nm Au were sequentially deposited on MgF_2_ layer; v) the patterned Au nanoantenna array was realized by ion beam etching. The device was stored in a dry and sealed environment before use.

### Preparation and Measurement of Poly(ethyl cyanoacrylate) Film

The thickness of the PECA film was controlled by the concentration of ECA in the acetone solution. The detailed preparation process is as follows: i) Acetone was used to adjust various concentrations of ECA; ii) 10 uL ECA solutions with different concentrations were dropped on the surface of the MPA with an area of 5 × 5 mm^2^; iii) The sample was spin‐coated for 15 s with a spin coater at a speed of 6000 rpm, and then protected by nitrogen and immediately measured in the experimental platform. The delay time between the spin‐coating and the beginning of FTIR measurement was about 5 s. The preparation of PECA film was carried out at 40% RH (relative humidity) and room temperature (average 26 °C). The thickness of the PECA film was measured using an AFM (Bruker Corporation). The SEM image was obtained with the Carl Zeiss ΣIGMA 500 SEM system at an acceleration voltage of 5 kV.

### Fourier Transform Infrared Measurements

The infrared spectrum of the device was collected using a FTIR spectrometer (IR Tracer‐100, Shimadzu) coupled to an infrared microscope with a liquid‐nitrogen‐cooled mercury cadmium telluride (AIM 900, Shimadzu). The numerical aperture of the infrared microscope was set to 0.4 and the objective x15. The signal acquisition area was limited in a single 100 × 100 µm^2^ array by the knife‐edge apertures. The measured spectrum of the Au mirror was used as the background spectrum. The settings of other parameters are: A mirror velocity of 40 kHz, resolution of 4 cm^−1^, and taking the average value after scanning 25 times for each measurement. A transparent plastic container with continuous injection of dry nitrogen was used to wrap the microscope to eliminate the interference of water vapor and CO_2_.

### Apparatus

Optical analyses were imaged by an optical microscope (Motic china group CO., Ltd. China). SEM analyses were obtained using a field emission SEM (Carl Zeiss ΣIGMA 500, Germany). The XPS spectra were obtained by an XPS (Thermo Scientific Escalab 250Xi). The film thickness analyses were performed by a commercial atomic force microscopy (Dimension Icon, Bruker Inc). The EDS analysis was carried out using a Bruker Quantax EDS system with an XFlash Silicon Drift Detector. Infrared spectral measurements were performed on a FTIR spectrometer (IRTracer‐100, Shimadzu) coupled to an infrared microscope with a liquid‐nitrogen‐cooled mercury cadmium telluride (AIM‐900, Shimadzu). Photolithography was performed by a Nikon I‐line stepper NSR‐2205 i‐12D. A magnetron sputtering system (FHR. Micro. 200, FHR Inc) was used to deposit Au and Ti layers.

### Statistical Analysis

Each experimental spectrum is the average of 25 scans. The LabSolution software (Shimazu Corporation, Japan) was used to preprocess the experimental spectrum data, including baseline calibration and normalization. Wavelength (*w*_l_, unit: µm) and wavenumber (*w*_n_, unit: cm^−1^) are converted by the formula: *w*_n_ = 10 000/*w*_l_. The coordinate parameters of the antenna in Figure S4, Supporting Information, were obtained using GetData Graph Digitizer software. The coordinate parameters of the PECA film thickness in Figures S8 and S12, Supporting Information, were obtained using the MATLAB program. The molecular vibration signal extraction, linear fitting, and data post‐processing are all completed by Origin (Origin‐Lab Corporation, USA) software.

## Conflict of Interest

The authors declare no conflict of interest.

## Supporting information

Supporting Information

## Data Availability

Research data are not shared.

## References

[1] K. Saha , S. S. Agasti , C. Kim , X. N. Li , V. M. Rotello , Chem. Rev. 2012, 112, 2739. 22295941 10.1021/cr2001178 PMC4102386

[2] J. F. Li , Y. J. Zhang , S. Y. Ding , R. Panneerselvam , Z. Q. Tian , Chem. Rev. 2017, 117, 5002. 28271881 10.1021/acs.chemrev.6b00596

[3] I. Lopez‐Lorente , B. Mizaikoff , Trac, Trends Anal. Chem. 2016, 84, 97.

[4] J. Mun , D. Lee , S. So , T. Badloe , J. Rho , Appl. Spectrosc. Rev. 2019, 54, 142.

[5] I. Alessandri , J. R. Lombardi , Chem. Rev. 2016, 116, 14921. 27739670 10.1021/acs.chemrev.6b00365

[6] R. Adato , H. Altug , Nat. Commun. 2013, 4, 2154. 23877168 10.1038/ncomms3154 PMC3759039

[7] D. E. Limaj , N. J. Wittenberg , D. Rodrigo , D. Yoo , S.‐H. Oh , H. Altug , Nano Lett. 2016, 16, 1502. 26761392 10.1021/acs.nanolett.5b05316

[8] J. Bochterle , F. Neubrech , T. Nagao , A. Pucci , ACS Nano 2012, 6, 10917. 23167482 10.1021/nn304341c

[9] D. Dregely , F. Neubrech , H. Duan , R. Vogelgesang , H. Giessen , Nat. Commun. 2013, 4, 2237. 23892519 10.1038/ncomms3237 PMC3731659

[10] K. Xu , Z. H. Ren , B. W. Dong , X. M. Liu , C. X. Wang , Y. H. Tian , C. K. Lee , ACS Nano 2020, 14, 12159. 32812748 10.1021/acsnano.0c05794

[11] H. Zhou , X. Hui , D. Li , D. Hu , X. Chen , X. He , L. Gao , H. Huang , C. Lee , X. Mu , Adv. Sci. 2020, 7, 2001173. 10.1002/advs.202001173 PMC7578855 33101855

[12] J. Wei , Y. Li , Y. Chang , D. M. N. Hasan , B. Dong , Y. Ma , C.‐W. Qiu , C. Lee , ACS Appl. Mater. Interfaces 2019, 11, 47270. 31769956 10.1021/acsami.9b18002

[13] Y. Chang , D. Hasan , B. Dong , J. Wei , Y. Ma , G. Zhou , K. W. Ang , C. Lee , ACS Appl. Mater. Interfaces 2018, 10, 38272. 30360088 10.1021/acsami.8b16623

[14] C. K. Chen , M. H. Chang , H. T. Wu , Y. C. Lee , T. J. Yen , Biosens. Bioelectron. 2014, 60, 343. 24836017 10.1016/j.bios.2014.04.019

[15] L. Kuehner , M. Hentschel , U. Zschieschang , H. Klauk , J. Vogt , C. Huck , H. Giessen , F. Neubrech , ACS Sens. 2017, 2, 655. 28723169 10.1021/acssensors.7b00063

[16] F. Neubrech , C. Huck , K. Weber , A. Pucci , H. Giessen , Chem. Rev. 2017, 117, 5110. 28358482 10.1021/acs.chemrev.6b00743

[17] F. Neubrech , A. Pucci , T. W. Cornelius , S. Karim , A. Garcia‐Etxarri , J. Aizpurua , Phys. Rev. Lett. 2008, 101, 157403. 18999639 10.1103/PhysRevLett.101.157403

[18] A. E. Cetin , S. Korkmaz , H. Durmaz , E. Aslan , S. Kaya , R. Paiella , M. Turkmen , Adv. Opt. Mater. 2016, 4, 1274.

[19] A. John‐Herpin , A. Tittl , H. Altug , ACS Photonics 2018, 5, 4117. 30828588 10.1021/acsphotonics.8b00847 PMC6390698

[20] D. Hasan , C. Lee , Adv. Sci. 2018, 5, 1700581. 10.1002/advs.201700581 PMC5978960 29876204

[21] K. Chen , R. Adato , H. Altug , ACS Nano 2012, 6, 7998. 22920565 10.1021/nn3026468

[22] T. D. Dao , K. Chen , T. Nagao , Nanoscale 2019, 11, 9508. 31049510 10.1039/c9nr00904c

[23] T. Yokoyama , D. T. Duy , K. Chen , S. Ishii , R. P. Sugavaneshwar , M. Kitajima , T. Nagao , Adv. Opt. Mater. 2016, 4, 1987.

[24] D. Rodrigo , A. Tittl , N. Ait‐Bouziad , A. John‐Herpin , O. Limaj , C. Kelly , D. Yoo , N. J. Wittenberg , S. H. Oh , H. A. Lashuel , H. Altug , Nat. Commun. 2018, 9, 2160. 29867181 10.1038/s41467-018-04594-x PMC5986821

[25] E. Aslan , S. Kaya , E. Aslan , S. Korkmaz , O. G. Saracoglu , M. Turkmen , Sens. Actuators, B 2017, 243, 617.

[26] H. Aouani , H. Sipova , M. Rahmani , M. Navarro‐Cia , K. Hegnerova , J. Homola , M. Hong , S. A. Maier , ACS Nano 2013, 7, 669. 23199257 10.1021/nn304860t

[27] E. Aslan , E. Aslan , R. Wang , M. K. Hong , S. Erramilli , M. Turkmen , O. G. Saracoglu , L. D. Negro , ACS Photonics 2016, 3, 2102.

[28] S. Gottheim , H. Zhang , A. O. Govorov , N. J. Halas , ACS Nano 2015, 9, 3284. 25727720 10.1021/acsnano.5b00412

[29] D.‐S. Su , D. P. Tsai , T.‐J. Yen , T. Tanaka , ACS Sens. 2019, 4, 2900. 31602973 10.1021/acssensors.9b01225

[30] F. Neubrech , S. Beck , T. Glaser , M. Hentschel , H. Giessen , A. Pucci , ACS Nano 2014, 8, 6250. 24811345 10.1021/nn5017204

[31] R. Adato , A. Artar , S. Erramilli , H. Altug , Nano Lett. 2013, 13, 2584. 23647070 10.1021/nl400689q

[32] J. Vogt , C. Huck , F. Neubrech , A. Toma , D. Gerbert , A. Pucci , Phys. Chem. Chem. Phys. 2015, 17, 21169. 25516198 10.1039/c4cp04851b

[33] Y. Y. Wang , C. Y. Chung , Y. P. Lee , J. Chem. Phys. 2016, 145, 154303. 27782495 10.1063/1.4964658

[34] H. Esen , G. Cayli , Eur. J. Lipid Sci. Technol. 2016, 118, 959.

[35] R. Kesarwani , A. Khare , Opt. Mater. 2019, 93, 98.

[36] G. Han , S. Kim , S. X. Liu , Polym. Degrad. Stab. 2008, 93, 1243.

[37] J. Ma , Z. L. Liu , B. B. Chen , L. L. Wang , L. P. Yue , H. S. Liu , J. J. Zhang , Z. H. Liu , G. L. Cui , J. Electrochem. Soc. 2017, 164, A3454.

[38] H. Zhou , C. Yang , D. Hu , S. Dou , X. Hui , F. Zhang , C. Chen , M. Chen , Y. Yang , X. Mu , ACS Appl. Mater. Interfaces 2019, 11, 14630. 30920795 10.1021/acsami.9b02087

[39] J. B. Wu , H. Shan , W. L. Chen , X. Gu , P. Tao , C. Y. Song , W. Shang , T. Deng , Adv. Mater. 2016, 28, 9686. 27628711 10.1002/adma.201602519

[40] J. Comyn , Int. J. Adhes. Adhes. 1998, 18, 247.

[41] A. Peppas , J. C. Wu , E. D. Vonmeerwall , Macromolecules 1994, 27, 5626.

[42] M. Fresta , G. Cavallaro , G. Giammona , E. Wehrli , G. Puglisi , Biomaterials 1996, 17, 751. 8730958 10.1016/0142-9612(96)81411-6

[43] S. Fan , W. Suh , J. D. Joannopoulos , J. Opt. Soc. Am. A 2003, 20, 569. 10.1364/josaa.20.000569 12630843

