# Supplementary material for: Multifunctional Chemical Sensing Platform Based on Dual‐Resonant Infrared Plasmonic Perfect Absorber for On‐Chip Detection of Poly(ethyl cyanoacrylate)
Source: Adv Sci (Weinh). 2021 Aug 22;8(20):2101879. doi: 10.1002/advs.202101879 (PMC8529490; doi:10.1002/advs.202101879)
Supplement: Supplementary file 1 — Supporting Information [file ADVS-8-2101879-s001.pdf]

© 2021 Wiley-VCH GmbH

ADVANCED SCIENCE
Open Access

Supporting Information

for *Adv. Sci.*, DOI: 10.1002/advs.202101879

Multifunctional Chemical Sensing Platform Based on Dual-Resonant Infrared Plasmonic Perfect Absorber for On-Chip Detection of Poly(ethyl cyanoacrylate)

Dongxiao Li, Hong Zhou, Xindan Hui, Xianming He, He Huang, Jiajia Zhang, Xiaojing Mu, Chengkuo Lee*, andYa Yang**

Copyright WILEY-VCH Verlag GmbH & Co. KGaA, 69469 Weinheim, Germany, 2021.

Supporting Information

Multifunctional Chemical Sensing Platform Based on Dual-Resonant Infrared Plasmonic Perfect Absorber for On-Chip Detection of Poly(ethyl cyanoacrylate)

Dongxiao Li, Hong Zhou, Xindan Hui, Xianming He, He Huang, Jiajia Zhang, Xiaojing Mu, Chengkuo Lee*, and Ya Yang**

Table of Contents

Note 1. Dual-resonant MPA Strategy 3

Note 2. Multiple Resonance Expansion.. 4

Note 3. SEM Micrograph of All Nine Kinds of Device 5

Note 4. Antenna size parameters come from SEM images 6

Note 5. EDS spectrum of PECA.. 7

Note 6. FTIR spectrum of PECA... 8

Note 7. Complex dielectric function of PECA 10

Note 8. SEIRA enhancement factor of MPA 13

Note 9. Chemical identification characteristics of dual-resonant MPA 16

Note 10. Simulated Near-Field Distribution of the X-Shaped Antenna Tip 19

Note 11. Sub-nm sensitivity of MPA platform 20

Note 12. Simulation Analysis of Measuring PECA Thickness 21

Note 13. Temporal Coupled Model Theory.. 22

References.. 29

Note 1. Dual-resonant MPA Strategy

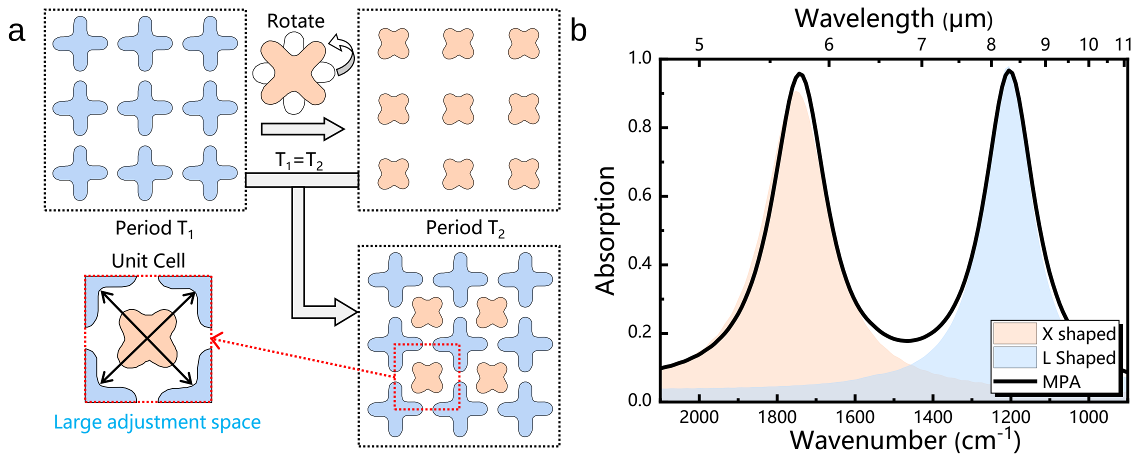

Figure S1. Dual-resonant MPA strategy. a) Schematic diagram of dislocation assembly of dual resonant array. b) Simulation of single and dual resonant array. The shaded areas represent the single resonance spectrum generated by the corresponding structure. The black curve represents the double resonance spectrum after dislocation assembly of the two structures. Clearly, the resonance of dual-resonant array is the superposition of the resonance of the two structures.

Note 2. Multiple Resonance Expansion

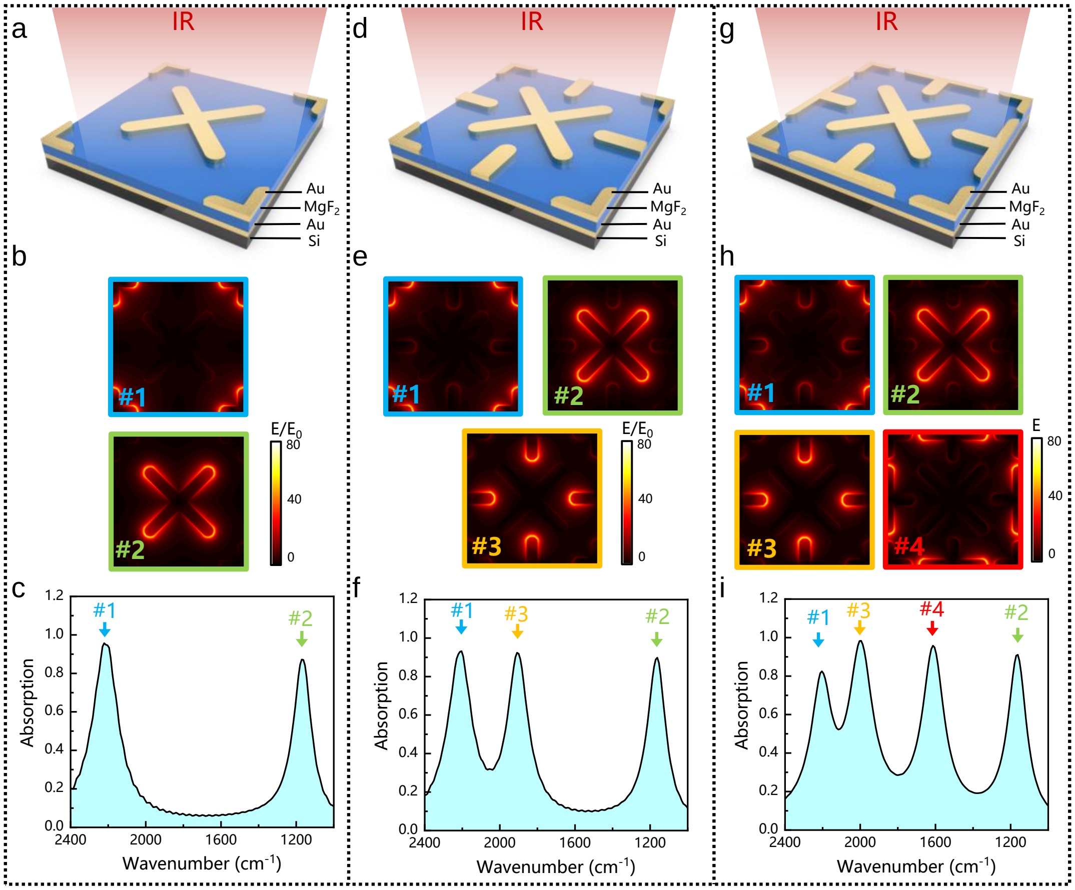

Figure S2. Schematic diagram of multiple resonance expansion. Antennas of different structures are assembled in a single unit cell to achieve multiple infrared resonance. The structural diagrams of the perfect absorber with (a) double resonance, (d) triple resonance, and (g) four resonance respectively. b-h) The simulated near-field intensity distribution of the corresponding multi-resonant absorber under fully polarized conditions. c-i) Simulated absorption spectra of the corresponding multi-resonant absorber.

Note 3. SEM Micrograph of All Nine Kinds of Device

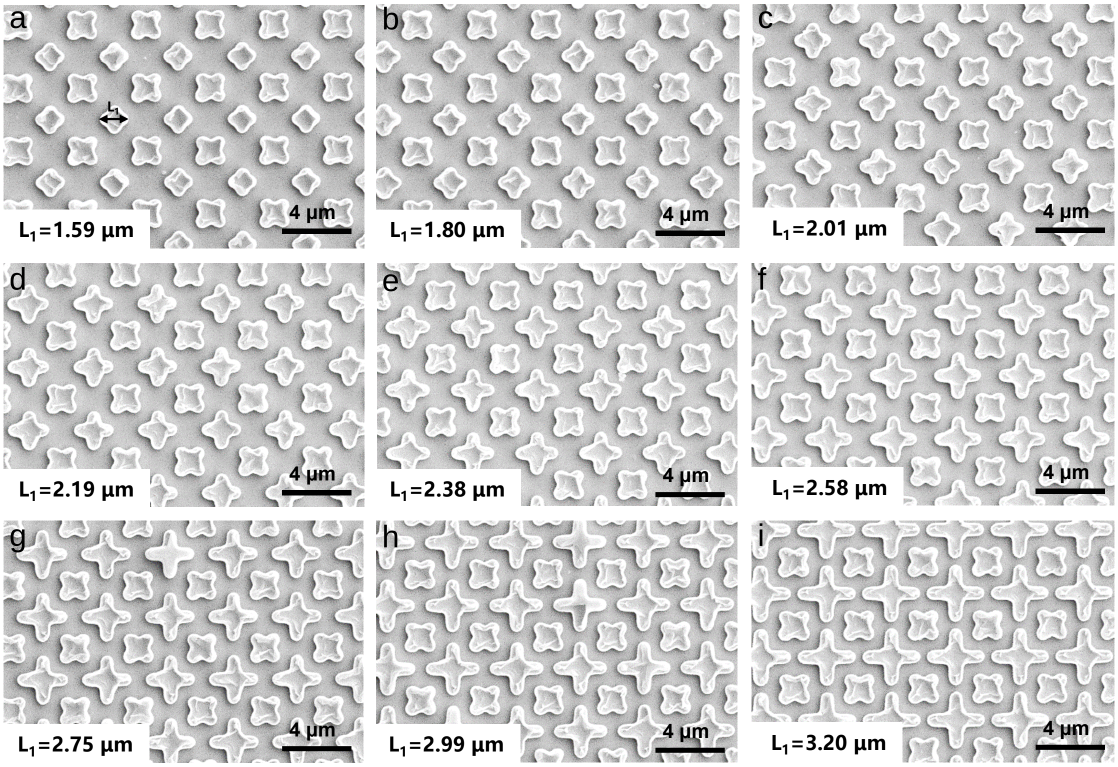

Figure S3. SEM micrograph of all nine kinds of device. SEM images of different L_1 lengths with a period of 3.6 μm. The L_1 lengths are varied from 1.59 to 3.20 μm.

Note 4. Antenna size parameters come from SEM images

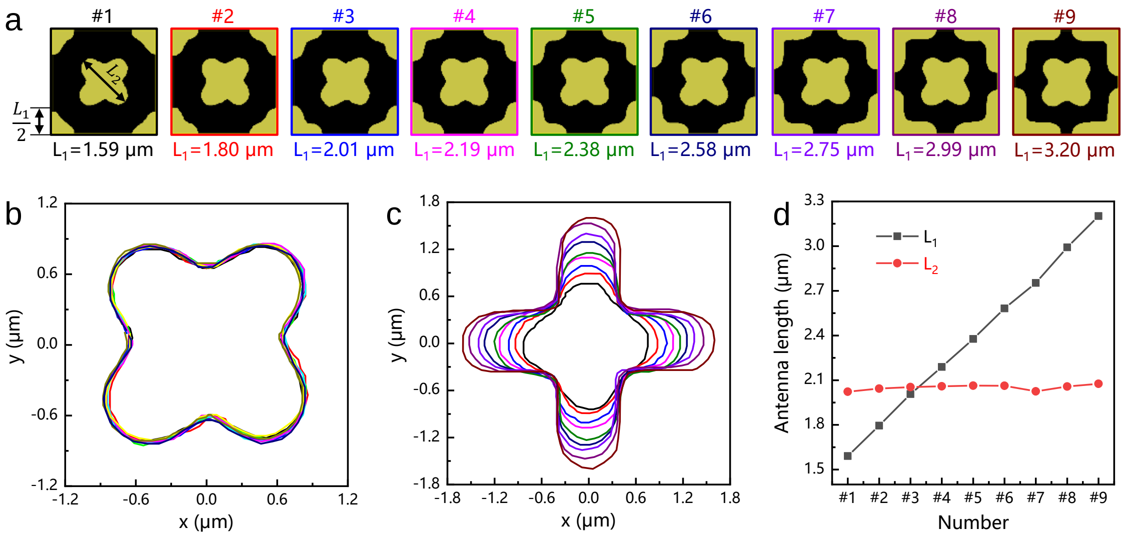

Figure S4. The size parameters of the antenna are extracted from the SEM image. a) Schematic diagrams of unit cell with different X-shaped/L-shaped antenna lengths. b, c) Extracted actual coordinate parameters of the X-shaped/L-shaped antenna from the SEM images. d) Arm length parameters for all nine antenna structures.

Note 5. EDS spectrum of PECA

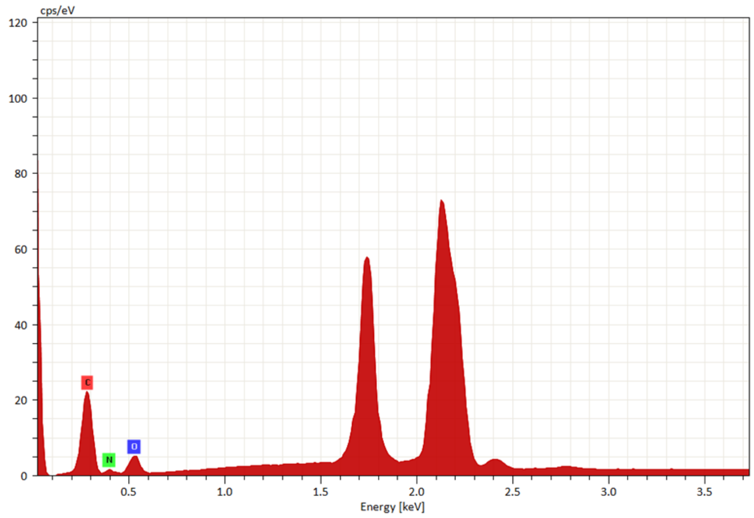

Figure S5. EDS spectrum of C, N, and O elements in PECA.

Note 6. FTIR spectrum of PECA

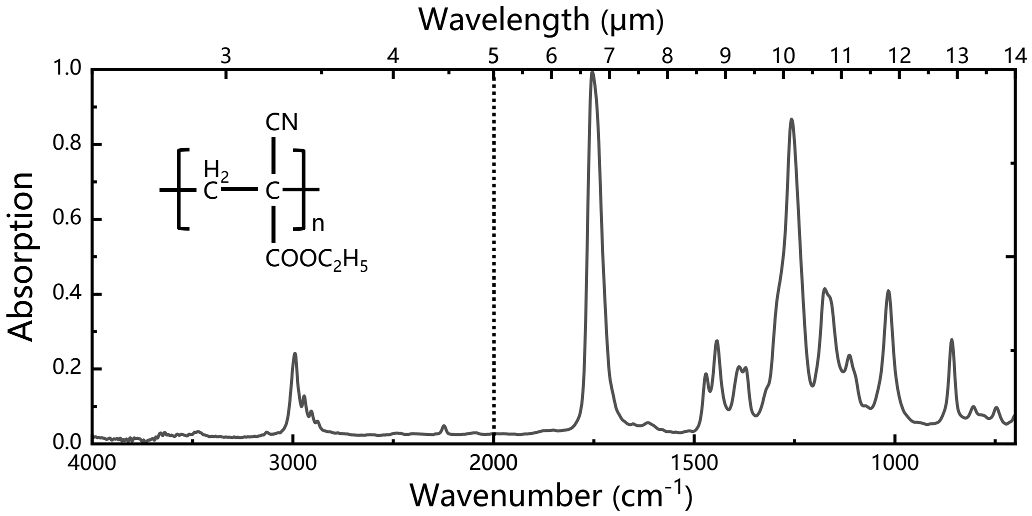

Figure S6. Measured FTIR spectrum of PECA. The spectrum used in this work is compressed in the range of 4000-2000 cm^{-1}. The inset shows the molecular structure of PECA.

Table S1 Major peak assignments for PECA at mid-IR.[1]

Wavenumber (cm^{-1})	Peak assignments and comments
3050-2800	C-H stretching vibrations (symmetric and asymmetric) of -CH2- and -CH3 groups
2248	-C≡N stretching vibration shifts
1747.5; 1015.7	-C=O stretching absorption
1615	-C=C- stretching vibration
1500-1350	-CH2 and -CH3 scissoring and bending region
1252.8	antisymmetric stretching vibration of C-O-C group

Note 7. Complex dielectric function of PECA

The complex dielectric function of a material largely determines its absorptivity, reflectivity and penetration depth. In classical physics, the complex dielectric function can be parameterized by Drude-Lorentz model,[2]

$$\varepsilon(\omega) = \varepsilon_\infty + \sum_{i=1}^{n} \frac{S_i}{(\omega_{pi}^2 - \omega^2 - j\omega\gamma_{pi})} \tag{S1}$$

where Lorentz oscillator is represented by three adjustable parameters: the oscillator resonance frequency w_{pi}, the damping frequency γ_{pi}, and the oscillator strength S_i. The parameter ε_∞ is the high frequency constant term.

According to the Fresnel formula, the reflectivity and dielectric function satisfy the following relationship under normal incidence,

$$R = \left| \frac{1-\sqrt{\varepsilon}}{1+\sqrt{\varepsilon}} \right|^2 \tag{S2}$$

According to the above equation (S1) and (S2), we can fit and obtain the complex dielectric function of the material. Here, we first obtain the reflectance spectrum of PECA through FTIR, and then fit the complex dielectric function of the PECA film through the Drude-Lorentz model. The fitting parameters are shown in Table S2, where ε_∞=2.07. According to equation (S1) and fitting parameters in Table S2, we can get the real and imaginary part of the complex dielectric of PECA, as shown in Figure S7a. To further verify the rationality of the fitting parameters, we imported the complex dielectric function of PECA into FDTD software. The simulation modeling is shown in the inset of

Figure S7b. 1 μm thick PECA film was set on the gold plate, and then the absorption spectrum of PECA through the monitor was obtained (Figure S7b). It can be seen that the simulated spectrum is in good agreement with the experimental spectrum (goodness of fit: R^2=0.91641), indicating the rationality of the fitting parameters.

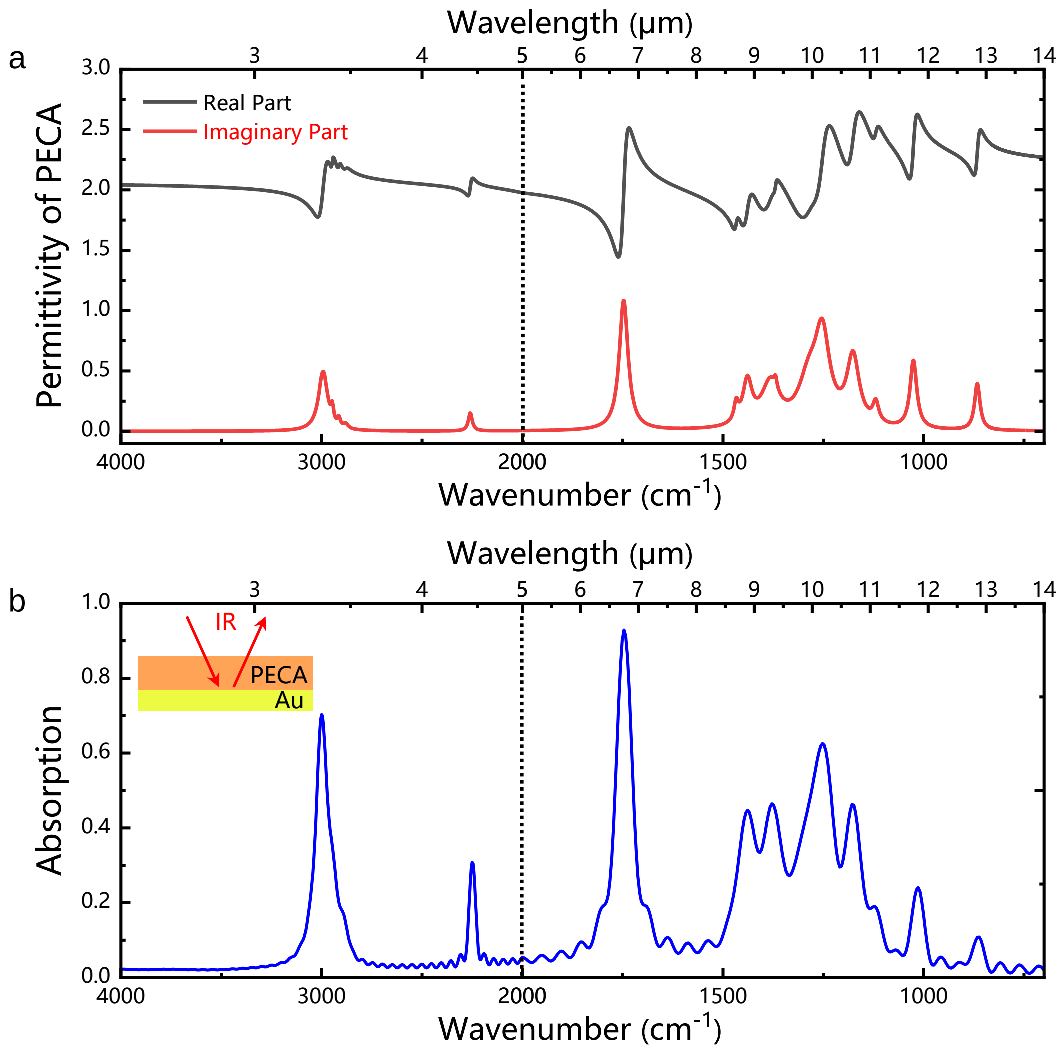

Figure S7. PECA complex permittivity and spectrum simulation. a) Real and imaginary part of PECA complex permittivity. b) Simulate the absorption spectrum of 1 μm thick PECA film on gold plate. The spectrum is compressed in the range of 4000-2000 cm^{-1}.

Table S2 Drude-Lorentz model fit complex permittivity parameters of PECA (ε_∞=2.07).

i	ω_{Pi} (cm^{-1})	S_i (cm^{-2})	γ_{pi} (cm^{-1})
1	2992.9	283.9	54.5
2	2946.6	69.4	13.7
3	2914.1	55.6	15.4
4	2880.1	55.8	28.5
5	2248.3	88.9	23.4
6	1747.5	223.6	26.5
7	1467.1	53.8	13.1
8	1439.3	120.0	27.9
9	1382.2	164.7	54.7
10	1369.1	29.8	8.1
11	1286.8	169.1	62.0
12	1252.8	200.2	44.7
13	1176.2	159.0	37.7
14	1119.4	58.4	18.1
15	1015.7	108.4	20.4
16	866.5	74.4	16.6

Note 8. SEIRA enhancement factor of MPA

The SEIRA enhancement factor (EF) of MPA coated with different film thicknesses are evaluated using the following relationship:

$$EF = \frac{I_{SEIRA}}{I_{ref}} \frac{N_{ref}}{N_{SEIRA}} \tag{S3}$$

Among them, I_{SEIRA} is the molecular vibration intensity derived from MPA coating PECA with different film thicknesses, I_{ref} is the molecular vibration intensity of 100 nm PECA film coated on Au plate. Both I_{SEIRA} and I_{ref} come from experimental data and refer to the maximum value of the relative absorption peak (Figure S8b). N_{ref} is the number of PECA molecules that contribute to absorption on the Au plate, and N_{SEIRA} is the number of PECA molecules that generate SEIRA signal. N_{ref} can be expressed as:

$$N_{ref} = P^2 t_{PECA} ni \tag{S4}$$

where P is the side length of the unit cell (P = 3.6 μm), t_{PECA} is the thickness of the PECA layer (t_{PECA} = 100 nm), n is the molecular number density, and i is the number of unit cells contained in the infrared beam area of 100×100 μm^2. N_{SEIRA} can be represented by the following relationship:

$$N_{SEIRA} = A_j (t_0 + t_1) ni \tag{S5}$$

Among them, the first term {$A_j(t_0+t_1)$} represents the volume of the PECA molecules at the tip of the metal antenna, t_0 is the thickness of the metal antenna (t_0=100 nm), and t_1 is the thickness of the PECA coated on the metal antenna (t_1=10, 20, 30, 60, 100 nm). A_j

represents the projected area of the molecules around the metal antenna on the *xy* plane, as shown in the orange area in Figure S8b. The area of the above orange area can be calculated in the commercial software Origin. The subscript *j* represents an X-shaped antenna or an L-shaped antenna. In each unit cell, a complete X-shaped antenna and a complete L-shaped antenna are included. Here, N_{SEIRA} consists of two parts: (1) the number of molecules that generate the SEIRA signal in a unit cell $\{A_j(t_0+t_1)n\}$, (2) the number of unit cells in the infrared beam region $\{i\}$. The product of the above two parts represents the total number of molecules that generate SEIRA signal in the infrared beam region. Finally, we obtained the enhancement factors of MPA for different PECA thicknesses, as shown in Figure 6f.

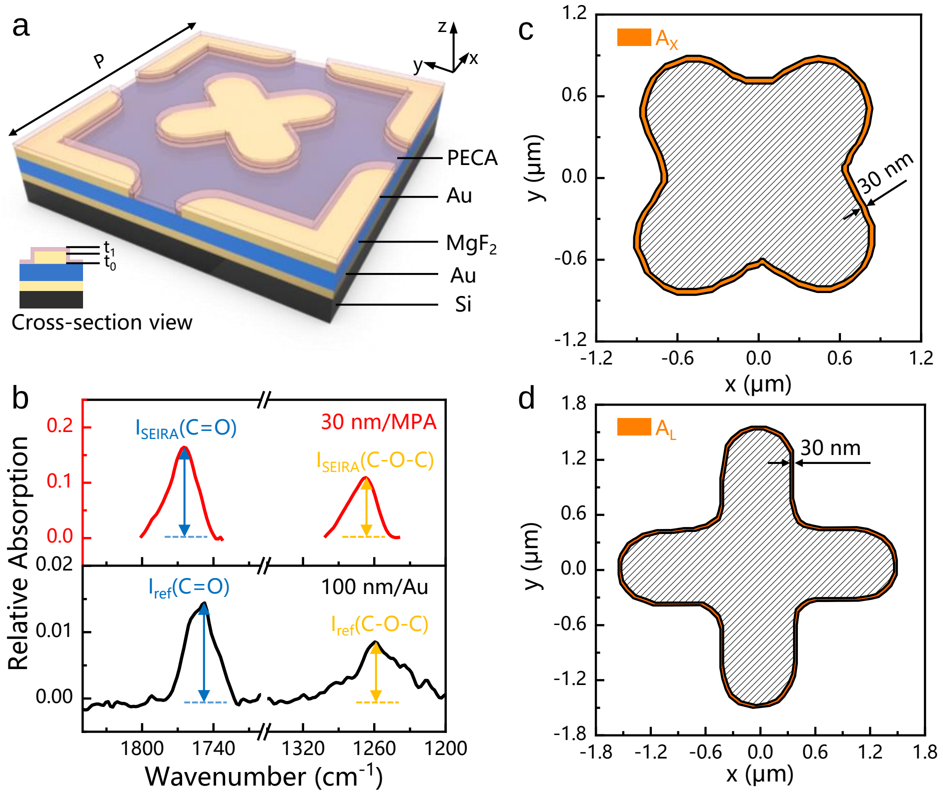

Figure S8. a) Schematic illustration of 30 nm PECA coated on MPA. b) The molecular intensity of I_{SEIRA} and I_{ref}. c, d) The projection of the X-shaped/L-shaped antenna (shaded area) and the surrounding PECA molecules (orange area) on the xy plane.

Note 9. Chemical identification characteristics of dual-resonant MPA

The proposed MPA has potential for chemical identification of trace amounts of molecules. Since the SEIRA enhancement is significant only when the vibration of the molecule matches the plasmonic resonance, the fingerprint of target molecule can be selectively detected by controlling the resonance wavelength of the MPA. In other words, MPA can selectively detect the fingerprint of the target molecule by controlling the resonance to achieve chemical identification. As a proof of concept, poly(ethylene imine) (PEI), polymethyl methacrylate (PMMA), and poly(ethyl cyanoacrylate) (PECA) are selected as analytes to investigate the chemical identification characteristics of MPA. The absorption spectra of the three analytes are shown in Figure S9a. Clearly, PEI has relatively concentrated fingerprint vibration peaks in the range of 1200cm^{-1} to 1600 cm^{-1}, and PMMA has a distinct peak near 1700 cm^{-1}. In addition, PECA has strong peaks at both 1750 cm^{-1} and 1250 cm^{-1}. The measured absorption spectra of the device covered by the three molecules are shown in Figure S9b. It can be seen by comparison that PEI can be identified with only a single resonance perfect absorber (blue curve in Figure S9b), which is due to the relatively concentrated fingerprint vibration peaks of PEI. However, a single resonance perfect absorber will lose its judgment when the fingerprint vibration peaks of the molecules are relatively dispersed, or two molecules have the same functional group. For example, it is difficult for a single resonance absorber to distinguish different chemical molecules when detecting very thin PMMA or PECA. This is because

both PMMA and PECA molecular structures have carbonyl (-C=O) fingerprint vibration peaks. Although the carbonyl center vibration frequency from PMMA and PECA is slightly different, the Fano effect produced by MPA-molecular coupling will cause the vibration frequency of the carbonyl group to move slightly, which further increases the difficulty of chemical identification of the single resonance absorber. Therefore, dual-resonant or multi-resonant absorbers will show great advantages in multi-fingerprint detection. Figure S9b shows the absorption spectra of dual-resonant MPA coated with PMMA (red curve) and PECA (black curve). It can be seen intuitively that the fingerprint vibration information detected by the second resonance peak will help the identification of chemical molecules when the analytes have the same functional group (such as a carbonyl group, the vibration peak is around 1750 cm^{-1}). In short, dual-resonance or multi-resonance absorbers have greater advantages in chemical molecular identification than single-resonant absorbers.

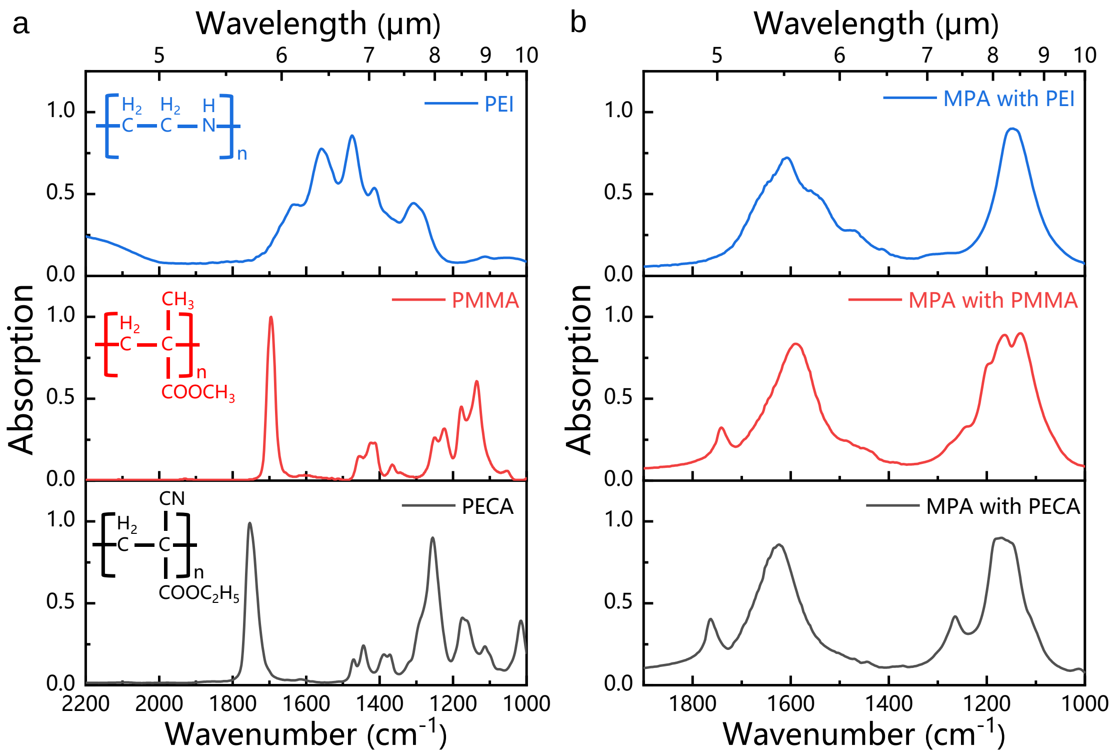

Figure S9. Selective detection characteristics of dual-resonant MPA. a) The measured absorption spectra of PEI, PMMA, and PECA. The insets show the chemical formula of the corresponding molecule. b) Absorption spectra of dual-resonant MPA coated with polymer molecules in (a). Obvious signals can be observed in the spectra only when the vibration of the molecule matches the plasmonic resonance.

Note 10. Simulated Near-Field Distribution of the X-Shaped Antenna Tip

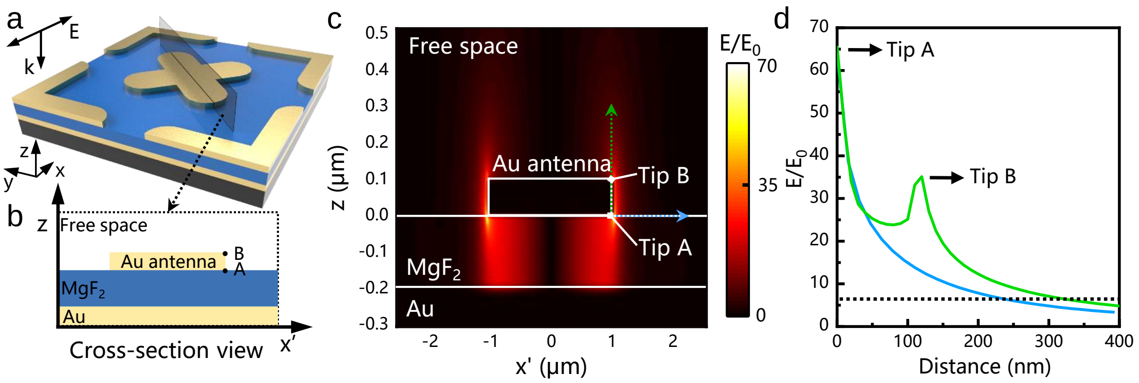

Figure S10. Simulated near-field distribution of the X-shaped antenna tip at the corresponding resonant frequency.

Note 11. Sub-nm sensitivity of MPA platform

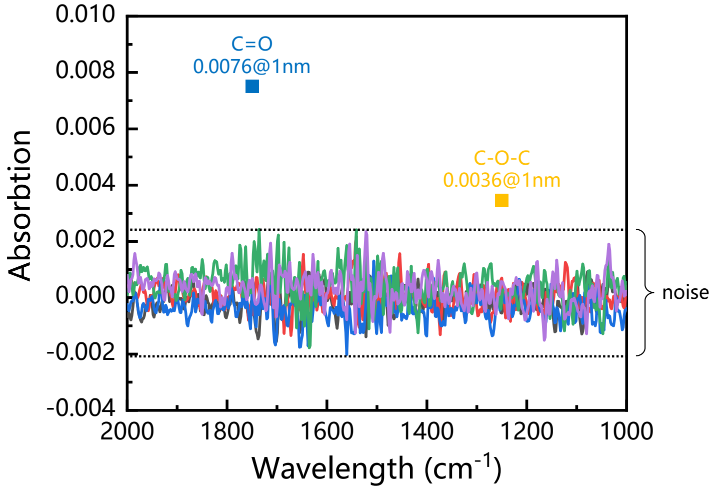

Figure S11. The background noise and maximum sensitivity of the MPA sensing platform.

Note 12. Simulation Analysis of Measuring PECA Thickness

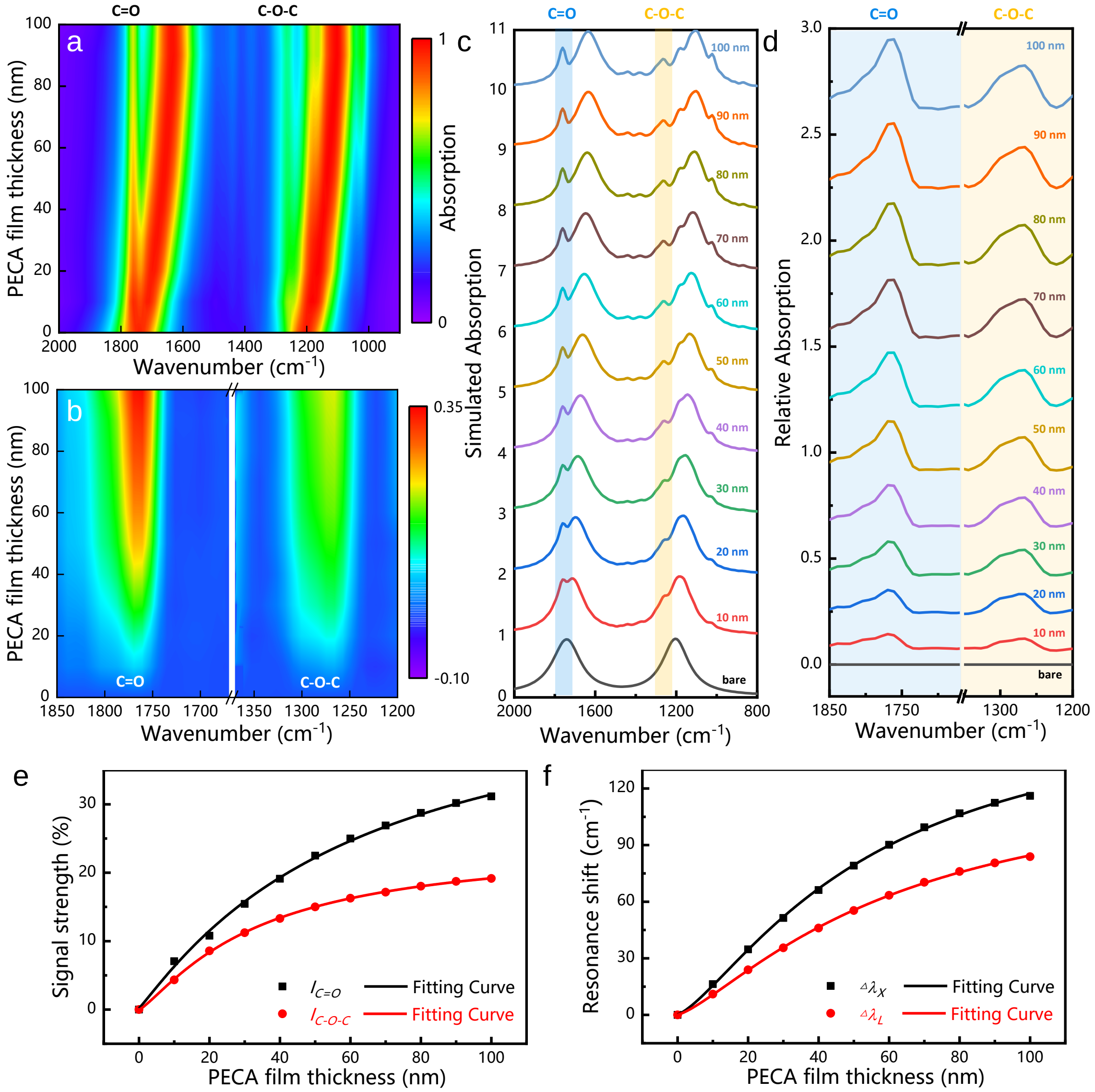

Figure S12. Simulation analysis of measuring PECA thickness using the dual-resonant MPA. a, c) Simulated absorption spectra of MPA covered with different thicknesses of PECA. The thickness varies from 10 nm to 100 nm. b, d) The corresponding relative absorption spectrum. e) The evolution of signal strength as a function of PECA film thickness. f) The evolution of resonance shift as a function of PECA film thickness.

Note 13. Temporal Coupled Model Theory

The temporal coupled model theory (TCMT) is derived from the classical mass spring system or circuit theory, and has been widely used in Fano resonance, polarization subsystem, electromagnetically induced transparency (EIT) and electromagnetically induced absorption (EIA).[3] Here, we use the TCMT model to understand the coupling characteristics between electromagnetic waves, metamaterials, and molecular vibrations. First, in the original framework of coupled mode theory, we analyze the dual-mode response characteristics of the device by adding a second resonant cavity. The generalized resonance model of MPA double resonance is shown in Figure S13. Here, S_{2+} and S_{2-} of port 2 are zero because the metamaterial absorber does not transmit light. In this case, TCMT can be described by the following equation:

$$\frac{da_1}{dt} = j\omega_1 a_1 - \gamma_1 a_1 + \kappa_1 S_{1+} + j\kappa_3 a_2 \tag{S6}$$

$$\frac{da_2}{dt} = j\omega_2 a_2 - \gamma_2 a_2 + \kappa_2 S_{1+} + j\kappa_3 a_1 \tag{S7}$$

$$S_{1-} = -S_{1+} + \kappa_1 a_1 + \kappa_2 a_2 \tag{S8}$$

Here, S_{1+} and S_{1-} refer to the input wave and the reflected wave, respectively, a_1 and a_2 are the mode amplitudes of the metamaterial resonator, ω_1 and ω_2 are the center frequencies of the metamaterial resonator, $\gamma_{1,2} = \gamma_{1o,2o} + \gamma_{1e,2e}$ is the damping rate, which is composed of the internal absorption loss rate ($\gamma_{1o,2o}$) and the external radiation loss rate ($\gamma_{1e,2e}$) of the harmonic oscillator. The coupling coefficient, κ_1 and κ_2 refer to the degree of coupling between the resonator and the incident wave S_{1+}. The coupling

coefficient $\kappa_{1,2}$ is not arbitrary. For single-port input and output, it is related to $\gamma_{1e,2e}$ and is restricted by $\kappa_{1,2}=\sqrt{2\gamma_{1e,2e}}$.[4] The parameter κ_3 is defined as the coupling strength between the antennas. For our device, the mutual coupling between the double resonances is very small, because they come from different structures and the resonance frequencies are far apart. To simplify the model, we assume that the coupling coefficient $\kappa_3 \approx 0$. According to the above three equations (S6)-(S8) can be used to determine the reflection, $R=\left|S_{1-}/S_{1+}\right|^2$, and absorption *A=1-R-T* (the transmission *T=0* for the absorber). Therefore, the absorption of the dual-resonant MPA can be written as

$$A = 1 - \left| 1 - \frac{2\gamma_{1e}}{j(\omega-\omega_1)+\gamma_{1o}+\gamma_{1e}} - \frac{2\gamma_{2e}}{j(\omega-\omega_2)+\gamma_{2o}+\gamma_{2e}} \right|^2 \tag{S9}$$

Furthermore, we established a model of the absorption spectrum of PECA-coated MPA by using TCMT. To simplify the model, we only consider resonator a_1 (mode amplitude comes from λ_X) coupled with C=O molecular vibration, and resonator a_2 (mode amplitude comes from λ_L) coupled with C-O-C molecular vibration. The schematic diagram is shown in Figure S14a. In the meantime, we assume that the dual resonances are independent of each other, i.e., the influence of the coupling coefficient κ_3 is ignored. Therefore, the dual resonance absorber and molecular interaction model is described by

$$\frac{da_1}{dt} = j\omega_1 a_1 - \gamma_1 a_1 + \kappa_1 S_{1+} + j\mu_{1b} b \tag{S10}$$

$$\frac{da_2}{dt} = j\omega_2 a_2 - \gamma_2 a_2 + \kappa_2 S_{1+} + j\mu_{2c} c \tag{S11}$$

$$\frac{db}{dt} = j\omega_b b - \gamma_b b + j\mu_{1b} a_1 \tag{S12}$$

$$\frac{dc}{dt} = j\omega_c c - \gamma_c c + j\mu_{2c} a_2 \tag{S13}$$

$$S_{1-} = -S_{1+} + \kappa_1 a_1 + \kappa_2 a_2 \tag{S14}$$

Here, the coupling coefficient $\kappa_{1,2} = \sqrt{2\gamma_{1e,2e}}$. The parameters $b(c)$, $\omega_b(\omega_c)$, $\gamma_b(\gamma_c)$ are the mode amplitude, center frequency and absorption damping of C=O (C-O-C) respectively, μ_{1b} (μ_{2c}) is the coupling rate between C=O (C-O-C) vibration and metamaterial cavity $a_1(a_2)$. Solving the set of equations: (S10)-(S14), the absorption A_2 of the coupled system can be written as

$$A_2 = 1 - \left| 1 - \frac{2\gamma_{1e}}{j(\omega-\omega_1)+\gamma_{1o}+\gamma_{1e}+\Gamma_b} - \frac{2\gamma_{2e}}{j(\omega-\omega_2)+\gamma_{2o}+\gamma_{2e}+\Gamma_c} \right|^2 \tag{S15}$$

where $\Gamma_b = \mu_{1b}^2 / \left[j\left(\omega-\omega_b\right)+\gamma_b \right]$ and $\Gamma_c = \mu_{2c}^2 / \left[j\left(\omega-\omega_c\right)+\gamma_c \right]$, $\omega_{1(2)}$ and $\omega_{b(c)}$ present the resonance frequency of the SEIRA device and the vibration frequency of the target molecule, respectively. Theoretically, $\omega_{1,2}$ and $\omega_{b,c}$ are determined by the SEIRA device and target molecule respectively, that is, the values of ω_1, ω_2 and ω_b, ω_c are fixed after the device is fabricated and the molecule is selected. Here, according to the experimental data, we set ω_1=1750.74 cm^{-1}, ω_2=1240.58 cm^{-1}, ω_b=1747.50 cm^{-1}, ω_c=1252.8 cm^{-1}. The fitting result of the measured spectral data calculated by using equation (S15) is shown in Figure S14b. There is an obvious difference between the fitted curve and the measured spectrum, including the disappearance of the PECA feature and the absorption far from unity. In equation S15, there are special values $\omega_{1,2}$ *and* $\omega_{b,c}$ corresponding to the device resonance and molecular vibration. However, the device resonance after the loading of molecules is at $\omega' = \omega_{1,2} - \Delta\omega_i$, which is not included in equation S15. Therefore, when we

use equation S15 to fit the device resonance at ω', a mismatch occurs, leading to the disappearance of the PECA feature and the absorption far from unity. In other words, it is because the resonant frequency of the plasmonic MPA is red-shifted in the presence of PECA, but the above equations ignore the effect of redshift.

In response to this issue, the researchers proposed to revalue ω based on the spectra of the device with the analyte. Although this method is effective to a certain extent, it leads to the lack of key information in the model, namely the redshift of the resonance. Furthermore, the update of ω means re-executing the entire fitting process, resulting in a decrease in efficiency. Here, we add an equation including redshift information to modify the model,

$$\omega_i' = \omega_{io} - \Delta\omega_i \qquad (i = 1, 2) \tag{S16}$$

where ω_i and ω_{io} represent the resonance frequency of the device before and after the analyte is covered, and $\Delta\omega_i$ is the redshift caused by the analyte. The modified absorption can be obtained by substituting equation (S16) into equation (S15),

$$A_3 = 1 - \left| 1 - \frac{2\gamma_{1e}}{j(\omega - \omega_1 + \Delta\omega_1) + \gamma_{1o} + \gamma_{1e} + \Gamma_b} - \frac{2\gamma_{2e}}{j(\omega - \omega_2 + \Delta\omega_2) + \gamma_{2o} + \gamma_{2e} + \Gamma_c} \right|^2 \tag{S17}$$

Figure S14c describes the fitting result of the measured spectral data calculated by using modified model, and an excellent fit was observed, which proves the validity of the modified model. Then, the modified model is used to further analyze the coupling relationship between MPA and PECA with different film thicknesses. The coupling parameters obtained from the fits are shown in Table S3, where R^2 is the goodness of fit.

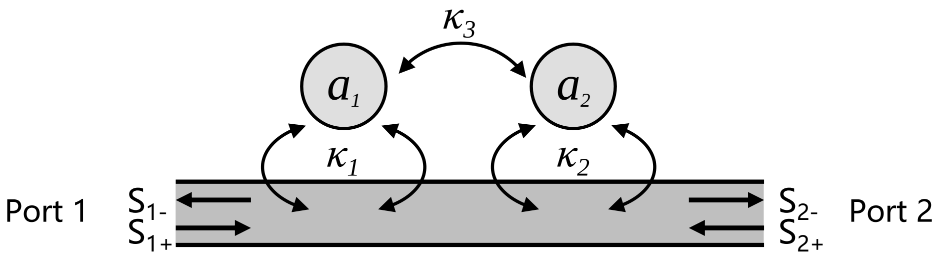

Figure S13. TCMT-based model for the dual-resonant MPA.

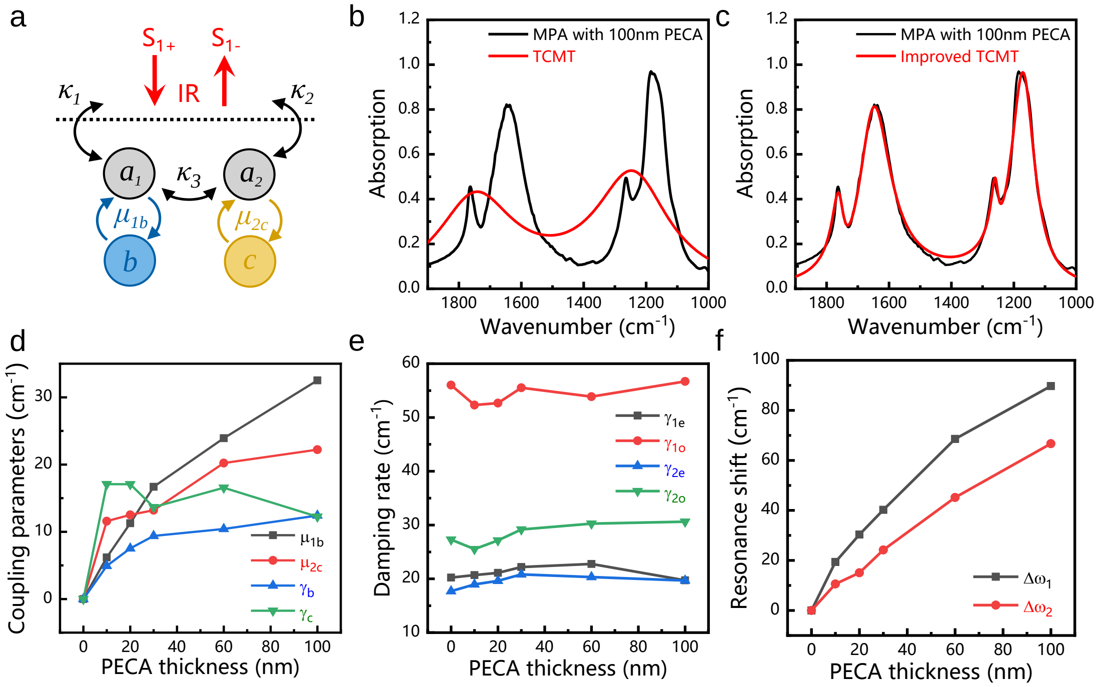

Figure S14. Near-field coupling in MPA-based sensing platform. a) Schematic illustration of the model including the PECA vibration mode based on TCMT. b) Curve fitting using an uncorrected TCMT-based model. c) Curve fitting using a corrected TCMT-based model. d) The coupling coefficient $\mu_{1b,2c}$ and damping $\gamma_{b,c}$ of PECA as a function of PECA thickness. e) Non-radiative ($\gamma_{1o,2o}$) and radiative damping rates ($\gamma_{1e,2e}$) as a function of PECA thickness. f) Resonance shift ($\Delta\omega_{1,2}$) as a function of PECA thickness.

Table S3 Model fit parameters for experimental spectra of MPA coated with PECA.

Parameters (unit: cm^{-1})	The thicknesses of PECA (unit: nm)					
	0	10	20	30	60	100
ω_1	1750.74	1750.74	1750.74	1750.74	1750.74	1750.74
ω_2	1240.58	1240.58	1240.58	1240.58	1240.58	1240.58
γ_{1e}	20.25	20.68	21.1	22.19	22.76	19.72
γ_{1o}	56.02	52.32	52.67	55.53	53.87	56.71
γ_{2e}	17.7	18.97	19.62	20.82	20.33	19.69
γ_{2o}	27.3	25.52	27.14	29.18	30.26	30.63
$\Delta\omega_1$	0	19.38	30.36	40.24	68.55	89.69
$\Delta\omega_2$	0	10.60	15.07	24.19	45.16	66.66
ω_b	0	1747.5	1747.5	1747.5	1747.5	1747.5
ω_c	0	1252.8	1252.8	1252.8	1252.8	1252.8
μ_{1b}	0	6.19	11.27	16.69	23.93	32.52
μ_{2c}	0	11.58	12.51	13.2	20.22	22.21
γ_b	0	4.92	7.52	9.38	10.42	12.41
γ_c	0	17.09	17.07	13.67	16.56	12.25
R^2	0.98581	0.98833	0.99153	0.98876	0.98484	0.98631

References

(1) S. K. Tomlinson, O. R. Ghita, R. M. Hooper, K. E. Evans, *Vib. Spectrosc.* **2006,** *40* (1), 133.

(2) J. Levallois, I. O. Nedoliuk, I. Crassee, A. B. Kuzmenko, *Rev. Sci. Instrum.* **2015,** *86* (3), 033906.

(3) H. A. Haus. *Waves and fields in optoelectronics.* Prentice-Hall, Inc., Englewood Clis, NJ, **1984**.

(4) R. Adato, A. Artar, S. Erramilli, H. Altug, *Nano Lett.* **2013**, 13, 2584.
